# Copy Number Gains at 8q24 and 20q11-q13 in Gastric Cancer Are More Common in Intestinal-Type than Diffuse-Type

**DOI:** 10.1371/journal.pone.0137657

**Published:** 2015-09-11

**Authors:** Dong-Hao Jin, Seong-Eun Park, Jeeyun Lee, Kyung-Mi Kim, Sung Kim, Duk-Hwan Kim, Joobae Park

**Affiliations:** 1 Department of Molecular Cell Biology, Samsung Biomedical Research Institute, Sungkyunkwan University School of Medicine, Suwon, 135–710, Korea; 2 Department of Internal Medicine, Samsung Medical Center, Sungkyunkwan University School of Medicine, Seoul, 135–710, Korea; 3 Department of Pathology, Samsung Medical Center, Sungkyunkwan University School of Medicine, 135–710, Seoul, Korea; 4 Department of Surgery, Samsung Medical Center, Sungkyunkwan University School of Medicine, Seoul, 135–710, Korea; National Cancer Center, JAPAN

## Abstract

The present study was aimed at discovering DNA copy number alterations (CNAs) involved in the carcinogenesis of stomach and at understanding their clinicopathological significances in the Korean population. DNA copy numbers were analyzed using Agilent 244K or 400K array comparative genomic hybridization (aCGH) in fresh-frozen tumor and matched normal tissues from 40 gastric cancer patients. Some of the detected CNA regions were validated using multiplex ligation-dependent probe amplification (MLPA) in six of the 40 patients and customized Agilent 60K aCGH in an independent set of 48 gastric cancers. The mRNA levels of genes at common CNA regions were analyzed using quantitative real-time PCR. Copy number gains were more common than losses across the entire genome in tumor tissues compared to matched normal tissues. The mean number of alterations per case was 64 for gains and 40 for losses, and the median aberration length was 44016 bp for gains and 4732 bp for losses. Copy number gains were frequently detected at 7p22.1 (20%), 8q24.21 (27%–30%), 8q24.3 (22%–48%), 13q34 (20%–31%), and 20q11-q13 (25%–30%), and losses at 3p14.2 (43%), 4q35.2 (27%), 6q26 (23%), and 17p13.3 (20%–23%). CNAs at 7p22.1, 13q34, and 17p13.3 have not been reported in other populations. Most of the copy number losses were associated with down-regulation of mRNA levels, but the correlation between copy number gains and mRNA expression levels varied in a gene-dependent manner. In addition, copy number gains tended to occur more commonly in intestinal-type cancers than in diffuse-type cancers. In conclusion, the present study suggests that copy number gains at 8q24 and 20q11-q13 and losses at 3p14.2 may be common events in gastric cancer but CNAs at 7p22.1, 13q34, and 17p13.3 may be Korean-specific.

## Introduction

Gastric cancer is the third leading cause of cancer deaths worldwide. Despite significant advances in the diagnosis and treatment of gastric cancer, five-year survival rates of gastric cancer patients remain below 30% in most countries [1]. In addition, approximately half of the patients who undergo curative surgical resection still develop loco-regional or distant metastases in spite of the multi-modality therapeutic approach and die from the disease [2,3]. Although most gastric cancers display similar clinical features, there is considerable heterogeneity in its histopathology and associated molecular changes [4]. Accordingly, it is important to identify molecular biomarkers involved in the carcinogenesis of gastric cancer for early detection and targeted therapy of the disease.

DNA copy number alteration (CNA) defined as DNA segments 1 kb or larger in size, is an important type of genetic alteration observed in cancer cells [5]. CNAs can influence gene expression, phenotypic variation and adaptation by disrupting proximal or distant DNA regulatory regions or by altering gene dosage levels [6,7]. In addition, the distribution of copy number is significantly different in distinct ancestral populations, which may result in different susceptibility to diseases across ancestral groups [8]. Recently, several groups have analyzed alterations of DNA copy number in gastric cancer using array comparative genomic hybridization (aCGH) and have identified novel genes important in the pathogenesis of gastric cancer [9–14]. For example, Tsukamoto et al. [10] investigated CNAs in 30 cases of gastric cancer by using BAC or PAC clones, and identified the most frequent regions of DNA copy number gains as 20q13, 20q11, 8q24, and 20p12, and those of losses as 4q34-qter, 5q12, 18q21, and 3p14. Fan et al. [11] detected CNAs in 64 gastric cancer tissues and 8 gastric cancer cell lines by using BAC clones, and observed that 20q12-20q13 and 9p21 were the most frequently amplified and deleted regions, respectively. In addition, Cheng et al. [12] studied CNAs in 27 gastric cancers by aCGH-244K and identified 8p11-q24, 20q11-q13, and 7q21-q22 as the most gained regions and 4q34, 6p25, 18q12, and 18q22 as the most lost regions. In these previous studies, various microarrays (BAC or PAC clone, oligo) were applied to investigate CNAs in gastric cancer, and the reported CNA regions were different for the various study populations.

To identify CNAs important in the pathogenesis of gastric cancer in the Korean population, we first performed a genome-wide analysis of DNA copy number using aCGH-244K or aCGH-400K in 40 gastric cancers and then validated the detected CNAs using a customized aCGH-60K in another set of 48 gastric cancers. The effects of CNAs on gene expression were analyzed in some of the genes with CNAs.

## Results

### Discovery of CNAs involved in the carcinogenesis of stomach

**Fig 1:**
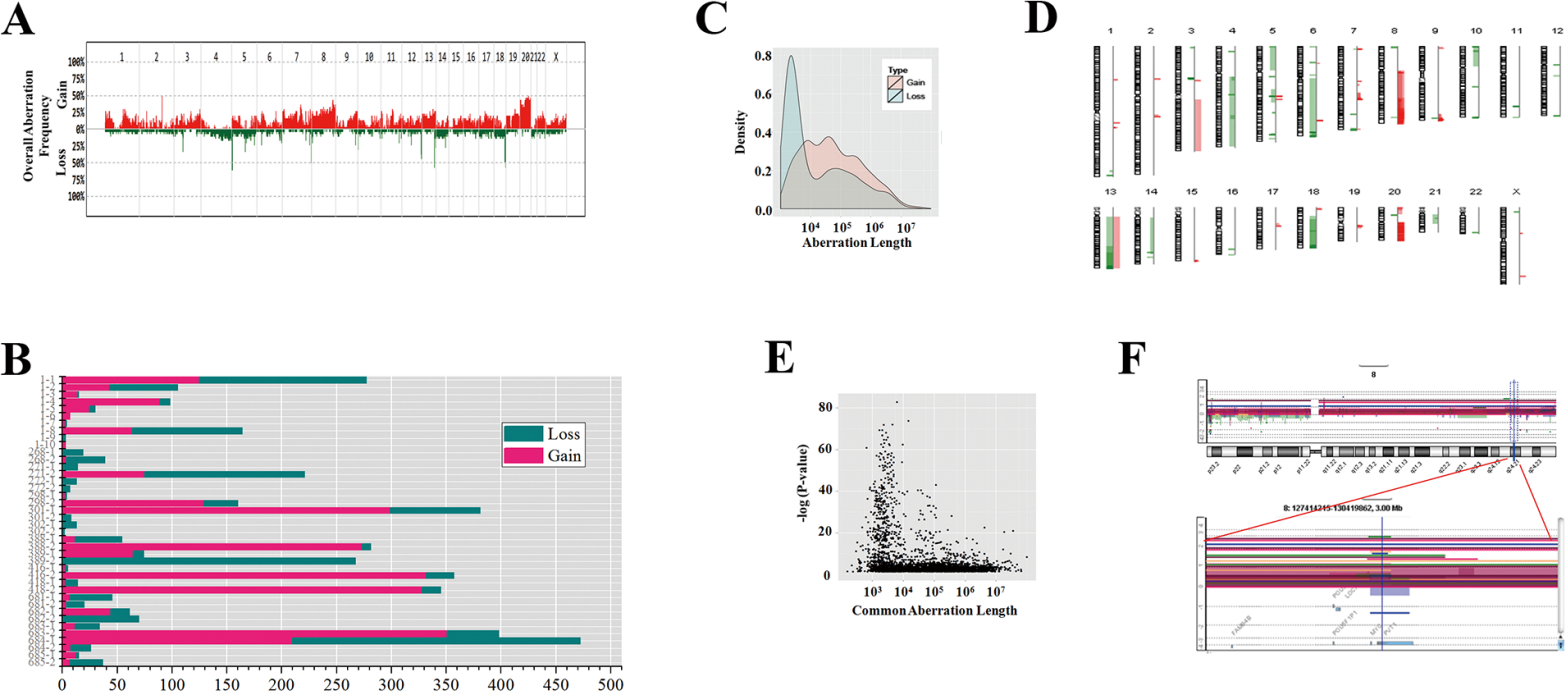
Copy number alterations of gastric cancer using aCGH. (A) Overall aberration frequency in gastric cancers is shown. The horizontal line represents chromosomes in order from 1 to 22 and X. The vertical line represents the frequency of gains and losses in all cases. (B) The number of aberrations in each case is shown. Magenta bars indicate gains and viridian bars indicate losses. (C) Aberration length—density plot is shown. (D) Common CNAs in 40 gastric cancer samples are shown. Within each chromosome, aberrations are expressed in order from the p telomere to q telomere. Reds on the right of the chromosomes indicate gains, and greens on the left of the chromosomes indicate losses. (E) Common aberration length with p-value plot is shown. (F) Common aberrations in chromosome 8 (upper panel) and aberrations around *MYC* gene at 8q24.21 (lower panel) are shown. Vertical lines indicate log_2_-based intensity ratio values, and each colored horizontal line represents a copy number alteration. Horizontal lines above the 0.25 of log_2_-based intensity ratio indicate samples with copy number gains. The large vertical blue bar in the lower panel indicates the center of the currently analyzed region.

To discover CNAs involved in the carcinogenesis of the stomach, tumor and matched normal tissues from 40 gastric cancer patients were analyzed using array comparative genomic hybridization (aCGH); 30 cases by aCGH-400K and 10 cases by aCGH-244K. CNAs were detected across the entire genome, and copy number gains were more common than copy number losses (Fig 1A). The number of CNAs was vastly different amongst individuals. The mean number of CNAs per case was 64 for gains and 40 for losses (Fig 1B), and the median length of the CNA region was 44016 bp for gains and 4732 bp for losses (Fig 1C). The common CNAs were detected using a context-corrected algorithm with a p-value threshold of 0.05 and overlap threshold of 0.9 (Fig 1D). Copy number gains were commonly detected on chromosomal regions 7p22.1, 8q24.21, 8q24.3, 13q34, and 20q11-q13, and copy number losses were frequently observed on 3p14.2, 6q26, 7q36.3, 13q34, and 18q23. The losses were largely detected at the ends of chromosomes, and the size was relatively small. The CNAs were less common on chromosomes 2 and 15. The common aberration lengths with a low p-value mainly fell within 1 kb-10 kb (Fig 1E). Common aberrations around *MYC* gene at 8q24.21 are shown in Fig 1F. The aCGH-244K and aCGH-400K data can be downloaded from the NCBI’s Gene Expression Omnibus portal (www.ncbi.nlm.nih.gov/geo) (accession number: GSE69318 and GSE69266, respectively).

### Validation of the aCGHs by MLPA analysis

**Fig 2:**
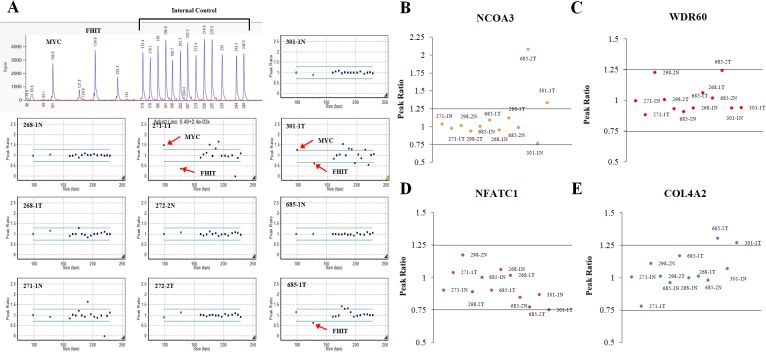
Multiplex Ligation-Dependent Probe Amplification (MLPA). (A) Upper left panel: representative image of capillary electrophoresis signals of MLPA analyzed by GeneMaker 2.0.0. Blue squares represent internal controls. Copy number alterations of *MYC* and *FHIT* were analyzed in 6 samples. (B-E) DNA copy number of *NCOA3* (B), *WDR60* (C), *NFATC1* (D), and *COL4A2* (E) were also analyzed using MLPA. Peak ratios within 1.25 ~ 0.75 was regarded as an indication of a normal DNA copy number, below 0.75 as an indication of a deletion, and above 1.25 as an indication of amplification. The T and N represent tumor and matched normal tissues, respectively.

Some genomic imbalances detected by the aCGH were validated using PCR-based multiplex ligation-dependent probe amplification (MLPA). Six of the 40 tissue samples analyzed using aCGH were available for MLPA. Copy number alterations of *MYC* (8q24.21), *FHIT* (3p14.2), *WDR60* (7q36.3), *COL4A2* (13q34), *NFATC1* (18q23), and *NCOA3* (20q12) were analyzed in six matched tumor and normal tissue pairs (268–1, 271–1, 272–2, 301–1, 685–1 and 685–2). *MYC* was amplified in 271-1T and 301-1T (Fig 2A), *NCOA3* was gained in 301-1T and 685-2T (Fig 2B), and *FHIT* was deleted in 271-1T, 301-1T, and 685-1T (Fig 2A). These results were highly consistent with those detected by aCGH. However, the copy number losses of genes such as *WDR60* (Fig 2C), *NFATC1* (Fig 2D), and *COL4A2* (Fig 2E), which were found to be lost in aCGH, were not detected in the MLPA. The length of the deletion region in the *WDR60* gene detected by aCGH was 2907 bp, and *COL4A2* and *NFATC1* were 1903 bp and 2596 bp, respectively. Based on these observations, it is likely that the aberrations spanning small regions observed by aCGH may be false.

### Additional validation of the CNA regions by aCGH-60K

To validate and narrow the CNA regions of recurrent (>20%) copy number gains or losses observed by aCGH in the 40 samples, we further analyzed their aberrations in tumor and matched normal tissues from another 48 gastric cancer patients using a customized aCGH-60K. The CNA regions at 8q24, 20q11-q13, 3p14.2, and 18q23 showed coincident alterations of copy number in the aCGH-60K, but CNAs at other regions, such as 20p13-20p12 and 20q21.2, did not show the same patterns as in the 244K and 400K, thereby suggesting heterogeneous results among aCGH platforms. To find minimal common regions (MCRs) of copy number alterations among the three platforms, we overlapped the CNA regions in a total of 88 samples. The MCRs of recurrent (>20%) copy number gains were detected on multiple chromosomal regions including 7p22.1 (20%), 7q22.1 (31%~44%), 8q24.21 (27%~30%), 8q24.3 (22%~48%), 13q34 (20%~31%), and 20q11~q13 (25%~30%) (Table 1 and Table A in S1 File). The MCRs of recurrent (> 20%) copy number losses were also found in seven chromosomal regions, including 3p14.2 (43%) harboring *FHIT* (Table 1 and Table B in S1 File).

**Table 1:** Minimal common regions^a^ of recurrent (>20%) copy number gains or losses.

Chromosomal regions	Frequency (N = 88)	Gene list^b^
**Gains**		
1p36.33	26%	SKI
1q42.13	28%	DUSP5P
2p25.3	35%	KIAA1106, MYT1L
5p15.33	27%	CCDC127
6p25.3	28%	EXOC2
6q21	43%	SCML4
6q25.3	32%	C6orf35, SLC22A1
7p22.1	20%	**FBXL18, ACTB, ACTG1, BC044606, DL492006, FBXL18, FSCN1,** RNF216
7q22.1	31%	DQ601342, PMS2L13, SPDYE3
7q22.1	44%	SPDYE2, SPDYE6
7q36.3	31%	PTPRN2
8p23.1	28%	AK307331, FLJ00326
8q24.13	25%	**TBIB1, BX648371**
8q24.21	27%	**DQ515898, DQ515899, LOC727677, POU5F1B, POU5F1P1**
8q24.21	30%	**BC042052, MYC**
8q24.3	48%	**AX748239, TRAPPC9**
8q24.3	23%	**CHRAC1, EIF2C2, MAPK15, SCRIB, ZNF707, PUF60, BOP1, RECQL4, SLC39A4, VPS28**
8q24.3	22%	**KIAA1688**
9q22.31	28%	PHF2
10p15.3	30%	DIP2C
10P15.3	52%	ADARB2, NCRNA00168
13q34	20%	QRTP1
13q34	31%	FLJ44054
13q34	22%	RASA3
16p13.3	24%	HMFN1876, LA16c-360B4.1, LMF1
18q21.1	34%	BC040860
19q13.12	22%	BC045185
20q11.21 –q11.22	25%	**ID1, NCRNA00028, PSIMCT-1, REM1, BCL2L1, SNTA1, E2F1** etc.
20q13.12	27%	**WFDC3, CTSA, UBE2C** etc.
20q13.12	28%	**CD40, CDH22, ETA2, NCOA3** etc.
20q13.31	30%	**RAE1, RBM38, ZBP1** etc.
20q13.33	27%	**CDH4**
20q13.33	28%	**ZBTB46, TPD52L2** etc.
**Losses**		
3p14.2	43%	FHIT
4q22.1	30%	FAM190A, KIAA1680
4q35.2	27%	**BC034307, BC038717, FAT1, MTNR1A, ZFP42** etc.
6q26	23%	PARK2, Parkin
17p13.3	22%	NXN
17p13.3	20%	**ABR,** NXN, **TIMM22**
17p13.3	22%	**TUSC5**
17p13.3	23%	**CRK, YWHAE**
18q21.1	20%	SMAD3
18q23	20%	**SALL3**

^a^ MCRs (Minimal Common Regions) were identified through the analysis of three arrays (aCGH-244K, aCGH-400K, aCGH-60K). MCR was defined as a 100 percent overlapping common region that was observed in all three arrays.

^b^ When CNAs occurred within whole DNA sequences of a gene, the gene is depicted as bold.

### Gene-dependent association between CNAs and mRNA levels

**Fig 3:**
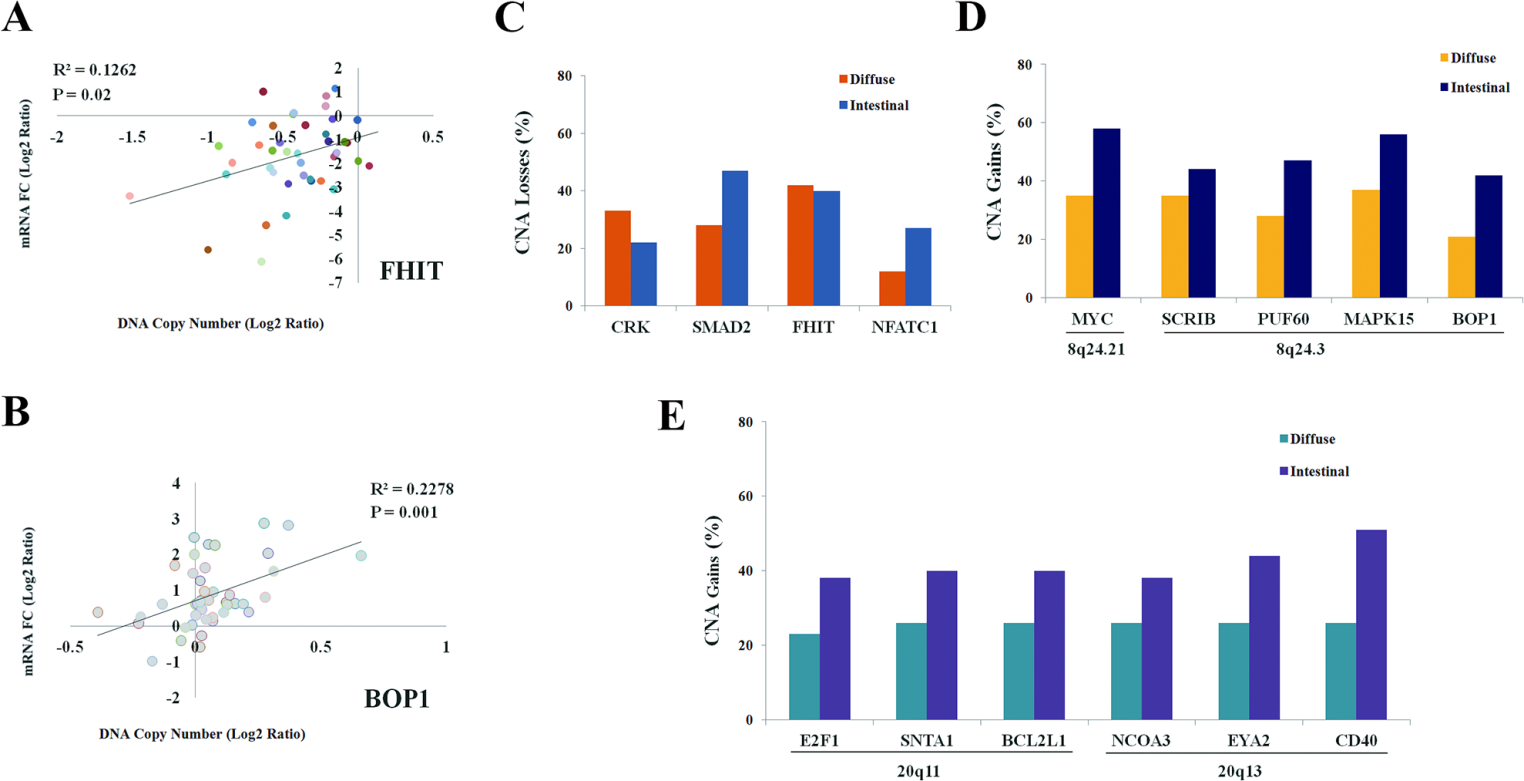
CNAs according to Lauren’s classification. (A-B) Correlations of log2 ratio of DNA copy number and mRNA fold change (FC) of *FHIT* (A) and *BOP1* (B) in tumor tissues compared to matched normal tissues were analyzed using Pearson’s correlation coefficients. Different color circles indicate different samples. (C-E) The differences in prevalence of copy number alterations of multiple genes were compared between diffuse-type cancers and intestinal-type cancers using Pearson’s chi-square test. Copy number gains of *MYC* (*P* = 0.03), *BOP1* (*P* = 0.03), and *CD40* (*P* = 0.01) occurred at a high prevalence in intestinal-type cancers compared to diffuse-type cancers. In general, copy number gains tend to occur more frequently in intestinal-type cancers than in diffuse-type cancers.

To investigate the effect of CNAs on gene expression, we measured mRNA levels of multiple genes (*MYC*, *SCRIB*, *PUF60*, *BOP1*, *SNTA1*, *E2F1*, *CD40*, *EYA2*, *NCOA3*, *FHIT*, *CRK*, and *SMAD2*) at the common aberration regions in 48 tumor and matched normal tissues and analyzed the association with the CNAs. The effect of CNAs on gene expression was analyzed by comparing the mRNA fold change (FC) in cancers with and without CNAs. The correlation between copy number alterations and corresponding gene expression was different according to copy number gains or losses. The majority of genes with copy number losses showed downregulation of mRNA: the mRNA level was downregulated in the *FHIT* (Table 2 and Fig 3A), *CRK*, and *SMAD2* genes (Table 2). However, we found the correlation between copy number gains and upregulation of mRNA levels was gene-specific: the mRNA levels in genes such as *MYC*, *PUF60*, *BOP1* (Fig 3B), and *E2F1* were positively associated with copy number gains. However, no association was found between mRNA levels and copy number gains of genes such as *SCRIB*, *BCL2L1*, *SNTA1*, *CD40*, *EYA2* and *NCOA3* (Table 2) suggesting that the relationship between copy number gains and expression may be gene-specific.

**Table 2:** The association of the expression of selected genes with copy number alterations.

Chromosomal regions	Gene	Total Number (Without CNA + with CNA)	mRNA FC	*P*-value^*^	mRNA FC without CNA	*P*-value^*^	mRNA FC with CNA	*P*-value^*^
**Gains**								
**8q24.21**	**MYC**	**26 (20 + 6)**	**Up**	**0.003**	**-**	**0.050**	**Up**	**0.010**
**8q24.3**	**SCRIB**	**26 (22 + 4)**	**Down**	**<0.001**	**Down**	**0.014**	**-**	**0.503**
**8q24.3**	**PUF60**	**26 (22 + 4)**	**Up**	**0.018**	**-**	**0.111**	**Up**	**0.038**
**8q24.3**	**BOP1**	**26 (22 + 4)**	**Up**	**<0.001**	**Up**	**<0.001**	**Up**	**0.024**
**20q11.21**	**SNTA1**	**26 (21 + 5)**	**Down**	**<0.001**	**Down**	**<0.001**	**-**	**0.202**
**20q11.21**	**E2F1**	**24 (20 + 4)**	**Up**	**<0.001**	**Up**	**<0.001**	**Up**	**0.030**
**20q13.12**	**CD40**	**26 (21 + 5)**	**Down**	**0.002**	**Down**	**0.010**	**-**	**0.147**
**20q13.12**	**CYA2**	**26 (21 + 5)**	**Down**	**<0.001**	**Down**	**<0.001**	**-**	**0.058**
**20q13.12**	**NCOA3**	**26 (21 + 5)**	**Down**	**<0.001**	**Down**	**0.002**	**-**	**0.177**
**Losses**								
**3p14.2**	**FHIT**	**25 (9 + 16)**	**Down**	**<0.001**	**Down**	**0.003**	**Down**	**<0.001**
**17p13.3**	**CRK**	**26 (13 + 13)**	**Down**	**<0.001**	**Down**	**<0.001**	**Down**	**<0.001**
**18q21.1**	**SMAD2**	**26 (16 + 10)**	**Down**	**<0.001**	**Down**	**<0.001**	**Dwon**	**<0.001**

* The significance of mRNA fold change (FC) of individual genes was statistically analyzed by one-sample t-test.

### Association of CNAs with clinicopathological characteristics

**Fig 4:**
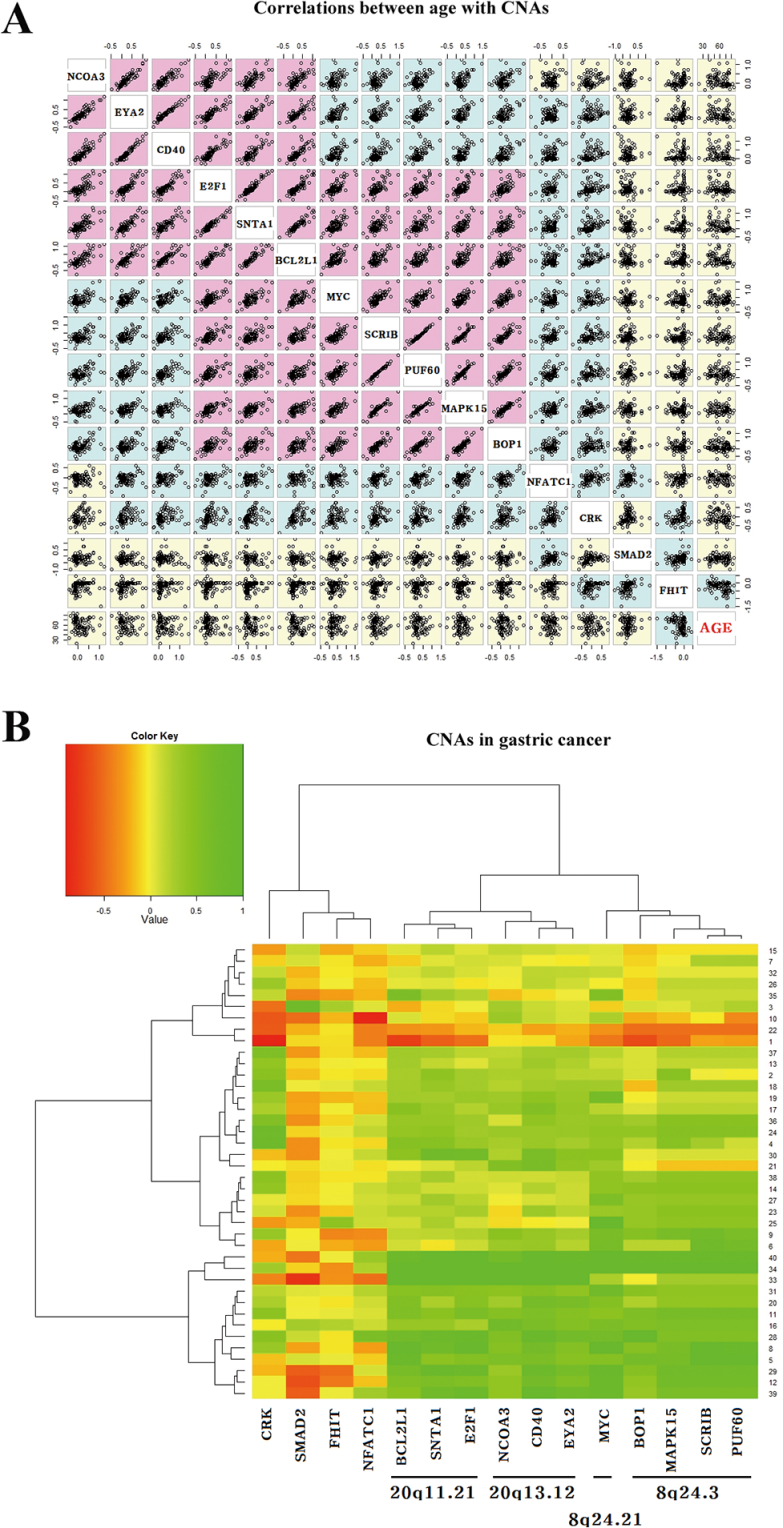
Correlations of CNAs levels with patient’s age at diagnosis and other CNAs in different chromosomal regions. (A) Correlations of CNAs levels with patient’s age were analyzed using Pearson’s correlation coefficient. Multiple tests were corrected using the Bonferroni correction. The Bonferroni-corrected *P*-values were calculated by multiplying the observed (uncorrected) *P*-values by the number of tested genes. Violet colors indicate Bonferroni-adjusted *P-*value < 0.05. (B) Unsupervised hierarchical clustering analysis of 15 genes in regions with recurrent (>20%) CNAs was performed to investigate correlations among CNAs levels of 11 genes showing copy number gains on 8q24.21, 8q24.3, 20q11.21, and 20q13.12, and 4 genes showing copy number losses. The numbers on the right side of the figure indicate patient identification number. The color scales indicate log2 intensity ratios of CNAs at individual gene. Value “zero (= log(2/2)” indicate a copy number of 2. Green and red colors represent copy number gain and loss, respectively.

The association between copy number alterations and clinicopathological variables was analyzed in 88 gastric cancer patients. Fifteen genes with recurrent (>20%) copy number alterations were selected for the analysis. Copy number losses of *CRK* (*P* = 0.07), *SMAD2* (*P* = 0.09), *FHIT* (*P* = 0.68), and *NFATC1* (*P* = 0.11) genes did not vary significantly between diffuse-type cancers and intestinal-type cancers (Fig 3C). However, copy number gains tended to occur at a high prevalence in intestinal-type cancers than in diffuse-type cancers (Fig 3D and 3E, Table C in S1 File). For *SCRIB* (*P* = 0.36), *PUF60* (*P* = 0.07), *MAPK15* (*P* = 0.08), *E2F1* (*P* = 0.14), *SNTA1* (*P* = 0.15), *BCL2L1* (*P* = 0.15), *NCOA3* (*P* = 0.22), and *EYA2* (*P* = 0.06), copy number gains occurred at a high prevalence in intestinal-type cancers than in diffuse-type cancers, but the difference was not statistically significant. Copy number gains of *MYC* (*P* = 0.03), *BOP1* (*P* = 0.03), and *CD40* (*P* = 0.01) were found at a significantly high prevalence in intestinal-type cancers compared to diffuse-type cancers. To detect age-related CNAs, we analyzed correlation between patient’s age and copy number change using Pearson’s correlation coefficients but found no correlation was found between copy number change of 15 genes and patient’s age (Fig 4A). Hierarchical clustering analysis was performed in order to group patients with similar CNAs. Most of the patients with copy number gains at 8q24 also had copy number gains at 20q11.21 or 20q13.12 (Fig 4B). Data were further divided into 4 clusters according to the presence of copy number gains at 8q24 and 20q11.21 (or 20q13.12). Copy number gains at 8q24 was significantly associated with copy number gains at 20q11.21 or 20q13.12 (*P* = 0.005, Fisher’s exact test; Table D in S1 File). These observations suggest that the two regions, 8q24 and 20q11.21 (or 20q13.12), may be similarly susceptible to copy number gains in gastric cancer.

## Discussion

The change of gene dosage by CNA is being increasingly recognized as an important component of tumorigenesis. To discover novel CNAs involved in the pathogenesis of gastric cancer, we performed a genome-wide analysis of CNAs in tumor and matched normal tissues from 88 gastric cancer patients and identified recurrent (> 20%) copy number gains at multiple chromosomal regions including 7p22.1, 8q24.21, 8q24.3, 13q34, 20q11~q13 and a recurrent losses at 3p14.2, 4q35.2, 6q26, and 17p13.3. The CNAs at 7p22.1, 13q34, and 17p13.3 have not been reported in other populations. The 7p22.1 regions identified in the present study contain the FBXL18, ACTB, ACTG1, and RNF216 genes. Although CNAs at 7p22.1 have not been reported in gastric cancer, several studies reported their impact on the development of ovarian clear cell adenocarcinoma [15] and endometriosis [16]. In this study, copy number alterations of *MYC* (8q24.21), *FHIT* (3p14.2), and *NCOA3* (20q12) were validated using MLPA, but copy number losses (*WDR60*, *COL4A2*, *NFATC1*) of around 2000bp were not validated by MLPA. We failed to perform extensive computational estimation of false positive rates of array-based calling. Instead, we have compared the prevalence of copy number losses between aCGH-244k & -400K and the aCGH-60K with highly dense probes according to the sizes of copy number losses: 1kb-5kb, 5kb-10kb, 10kb-50kb, 50kb-100kb, and 100k-. Statistically significant differences were found only in the copy number losses of small size (1kb-5kb) (Table E in S1 File). In addition, the significant differences were found in chromosomal locus-specific manner: no differences were found in chromosomes 3, 6, 16, 17, and 20 (data not shown). Therefore, it is possible that copy number losses of small size detected in aCGH-244K and aCGH-400K may be false in some loci.

We furthermore analyzed minimal common regions of recurrent (≥10%) amplification or deletion in 88 gastric cancers (Table F in S1 File) and compared them with the large gastric cancer TCGA (The Cancer Genome Atlas) study (14) and three previous studies (Table G in S1 File). The TCGA study was comprised of 295 primary gastric adenocarcinomas and identified 30 focal amplifications and 45 focal deletions. Amplification (≥ 5 copies) at 8q24.21 (*MYC*), 17q12 (*ERBB2* etc.), 20q11.1-q13.33 *(EYA2*, *NCOA3* etc.), and deletion (0 copies) at 3p14.2 (*FHIT*) were observed in our data as well as the data from the TCGA and others’ studies (Table G in S1 File). However, CNAs at 7p22.1, 13q34, and 17p13.3 have not been reported in the TCGA study and other populations. The number of the regions of the CNAs identified in the TCGA study was larger than the present study, which might result from the different subgroups of sample members. The TCGA study consists of larger intestinal-type (66.4%) compared to diffuse-type cancers (23.4%).

Among the genes located on 8q24.21, MYC is known to promote the growth and proliferation of normal gastric cells, and knockdown of *MYC* restrains the growth and proliferation of gastric cancer cells [17]. *MYC* encodes a transcriptional factor that regulates a variety of genes related to proliferation, differentiation, and apoptosis [18]. *MYC* is amplified and over-expressed in gastric cancer [19], and its expression increases progressively as the cancer develops [20]. *MYC* amplification is associated with the aggressive behavior of gastric cancer cells [21,22]. In this study, copy number gains of *MYC* were found at a high prevalence in the intestinal-type cancers as compared to the diffuse-type cancers, supporting the observation that MYC protein expression is more frequently observed in intestinal-type tumors than in diffuse-type tumors [23]. We have analyzed the effect of *MYC* CNAs on overall survival within each type. Patients with copy number gains of *MYC* had poor overall survival compared to those without, but the difference was not statistically significant in diffuse type and intestinal type cancers (S1 Fig). The copy number gains of the *POU5F1B* (POU domain class 5 transcription factor 1B) pseudogene on 8q24.21 were found in 27% of the samples analyzed. *POU5F1B* is known to be associated with mRNA abundance and an aggressive phenotype in gastric cancer [24].

The 8q24.3 and 20q11-q13 regions contain hundreds of genes (Table A in S1 File), but many are unlikely involved in oncogenesis. Among the genes located in these regions, we analyzed the mRNA levels of *SCRIB*, *PUF60*, and *BOP1* at 8q24.3 and *SNTA1*, *E2F1*, *CD40*, *EYA2*, and *NCOA3* at 20q11-q13 (Table 2). In the present study, copy number gains of *SCRIB*, *SNTA1*, *CD40*, *EYA2*, and *NCOA3* were not associated with a fold change in mRNA levels. However, the *PUF60*, *BOP1*, and *E2F1* genes were found to be significantly over-expressed in tumor tissues with copy number gains. *PUF60* was over-expressed in cancers with CNAs (*P* = 0.038), but its expression was not significantly different between tumor tissues and matched normal tissues in samples without CNAs (*P* = 0.111). PUF60 (poly-U binding splicing factor 60kDa), a FUSE-binding protein-interacting repressor (FIR), plays a role in nuclear processes such as pre-mRNA splicing and transcriptional regulation. In addition, PUF60 suppresses *MYC* transcription at the P2 promoter through the core-TFIIH basal transcription factor [25]. Recently, Gumireddy et al. [26] reported that PUF60 is required for the regulator function of translational regulatory IncRNA (treRNA), which is involved in tumor invasion and metastasis. Copy number gains of *PUF60* show a strong positive correlation with expression in gastric cancer [27] and in ovarian cancer [28]. These observations suggest that copy number gains of *PUF60* may be a major mechanism underlying the over-expression of the gene in gastric cancer.

In contrast to PUF60, the BOP1 and E2F1 were found to be over-expressed in tumor tissues with copy number gains as well as in those without. Copy number gains of *BOP1* and *E2F1* in this study occurred in 23% and 25% of samples studied, respectively. Increased mRNA fold change of *BOP1* was significant in tumor tissues with copy number gains (*P* = 0.024) as well as in those without (*P* < 0.001). BOP1 (block of proliferation 1) is a component of the PeBoW (Pes1, Bop1, and WDR12) complex, which is required for maturation of 28S and 5.8S ribosomal RNAs and formation of the 60S ribosome [29]. BOP1 plays an oncogenic role in hepatocellular carcinoma by inducing epithelial-mesenchymal transition (EMT) and promoting actin cytoskeleton remodeling [30]. The *BOP1* gene is known to be over-expressed in rectal cancer with 8q gain [31], and dosage increase of the *BOP1* gene is associated with an increase of *BOP1* mRNA in colorectal cancer [32]. The E2F1 was also over-expressed in tumor tissues with copy number gains (*P* < 0.001) and in those without (*P* = 0.03). E2F1 plays a crucial role in the control of the cell cycle and its activity is regulated through binding to retinoblastoma protein in a cell-cycle-dependent manner. Over-expression of E2F1 is associated with the development of a variety of tumors, and the increased copy number of *E2F1* is known to be associated with over-expression of the gene in melanoma [33] and cervical cancer [34]. Based on these observations, it is likely that the overall impact of copy number gains on gene expression in gastric cancer varies in a gene-dependent manner.

Although copy number gains at 13q34 were not reported in gastric cancer, the gains were found in 20–30% of samples studied. Copy number gains at 13q34 are known to be associated with the progression of cervical intraepithelial neoplasia to squamous cell carcinoma [35] and with small bowel adenocarcinoma [36]. Copy number gains at 17q12 are frequent in gastric cancer. In the present study, several genes, including *ERBB2*, *GRB7*, *STARD3*, *PPP1R1B*, *RARA*, and C17orf37, were amplified in 15–20% of the 88 cases, consistent with other studies [37,38]. We did not evaluate the correlation of copy number and expression levels of the genes, but several groups have reported that the genes are important in the development of gastric cancer. Among them, *ERBB2 (HER2)* is frequently amplified and over-expressed in gastric cancers [39–41], and amplification of *HER2* was strongly associated with poor survival, particularly in the intestinal type of gastric cancer [42]. Immunoreactivity of ERBB2 also occurs at a higher prevalence rate in intestinal type than in the diffuse subtypes [43]. Furthermore, the *PPP1R1B-STARD3* fusion transcript in human gastric cancer increases colony formation through the activation of phosphatidylinosil-3-kinase and AKT signaling [44]. Frequent amplification of *GRB7* and positive changes in expression were also reported in gastric cancer [41,43].

The most frequent losses in this study were detected on 3p14.2 (39% in diffuse-types and 37% of intestinal types), where *FHIT* is located. *FHIT* is a well-known tumor suppressor gene [45], and is often involved in the loss of heterozygosity (LOH) and deletions in human tumors [46]. Primary gastric carcinomas represent a rearrangement of the *FHIT* gene and 20 of 30 (67%) samples exhibited an absence of *FHIT* protein expression [47]. Loss of *FHIT* protein expression correlates with disease progression and poor differentiation in gastric cancer [48]. In the present study, we observed that *FHIT* expression was reduced in gastric cancers with or without its CNA, suggesting that gene dosage as well as other mechanisms regulate *FHIT* expression in gastric cancer. A somatic missense mutation (exon 6, codon 61, ACG → ATG) of *FHIT* has also been identified in gastric cancers [49]. Furthermore, a high frequency of promoter hypermethylation of *FHIT* (62%) is observed in gastric cancers [50]. Therefore, integrating copy number data with additional genomic data is essential to comprehensively understanding the genetic control of gene expression [51].

Copy number losses of several genes in this study were not significantly different between diffuse-type cancers and intestinal-type cancers. However, the prevalence of copy number gains was different between both types in certain genes, suggesting that environmental factors may be more influential in copy number gains than losses. In addition, patients with copy number gains on 8q24.21 and 8q24.3 tended to have gains on 20q11-q13, suggesting both regions may be equally susceptible to copy number variation. This study was severely limited due to the small number of samples and the lack of survival data. Further study in a large cohort is required to understand the functional significance of CNAs discovered in this study. In addition, mRNA measurements were not performed at a genome level. We analyzed relationship between mRNA levels of some genes known to be important in the pathogenesis of human cancer and the CNAs. A significant correlation was found between the expression levels of *MYC*, *PUF60*, *BOP1*, and *E2F1* genes and their CNAs (Table 2). A statistically significant correlation between CNAs of *MYC*, *PUF60*, and *E2F1* genes and their expression levels was also found by Fan et al. (11). However, further study is required to clearly understand the effect of CNAs on gene expression. In conclusion, the present study suggests that DNA copy number gains at 8q24.21, 8q24.3, 20q11-20q13 and losses at 3p14.2 may be common events in gastric cancer. However, CNAs at 7p22.1, 13q34, and 17p13.3 may be Korean-specific. In addition, copy number gains may be more frequent in intestinal-type than diffuse-type gastric cancer.

## Materials and Methods

### Study population and DNA extraction

A total of 88 patients, 35 women and 53 men, who had undergone curative surgical resection for gastric cancer between November 2004 and October 2010 at the Department of Surgery in the Samsung Medical Center, Seoul, Korea, participated in this study. Surgically removed tumor tissues were collected after obtaining written informed consent from all of the patients. This study was approved by the Samsung Medical Center (SMC) Institutional Review Board (IRB). The tumors were snap-frozen in liquid nitrogen and stored at −80°C until needed. Prior to DNA extraction from the fresh frozen tissues, the sections were placed on slides and stained with H&E to evaluate the admixture of tumorous and non-tumorous tissues. Tumor and non-tumor areas were microdissected carefully under a microscope. The microdissected tissues were digested with proteinase K, and the genomic DNA was isolated according to the instructions of the manufacturer (DNeasy Tissue kit, Qiagen, Valencia, CA). The sample consisted of 43 diffuse-type cancers, 41 intestinal-type, and 4 mixed-type cancers.

### CNA analysis using aCGH

**Fig 5:**
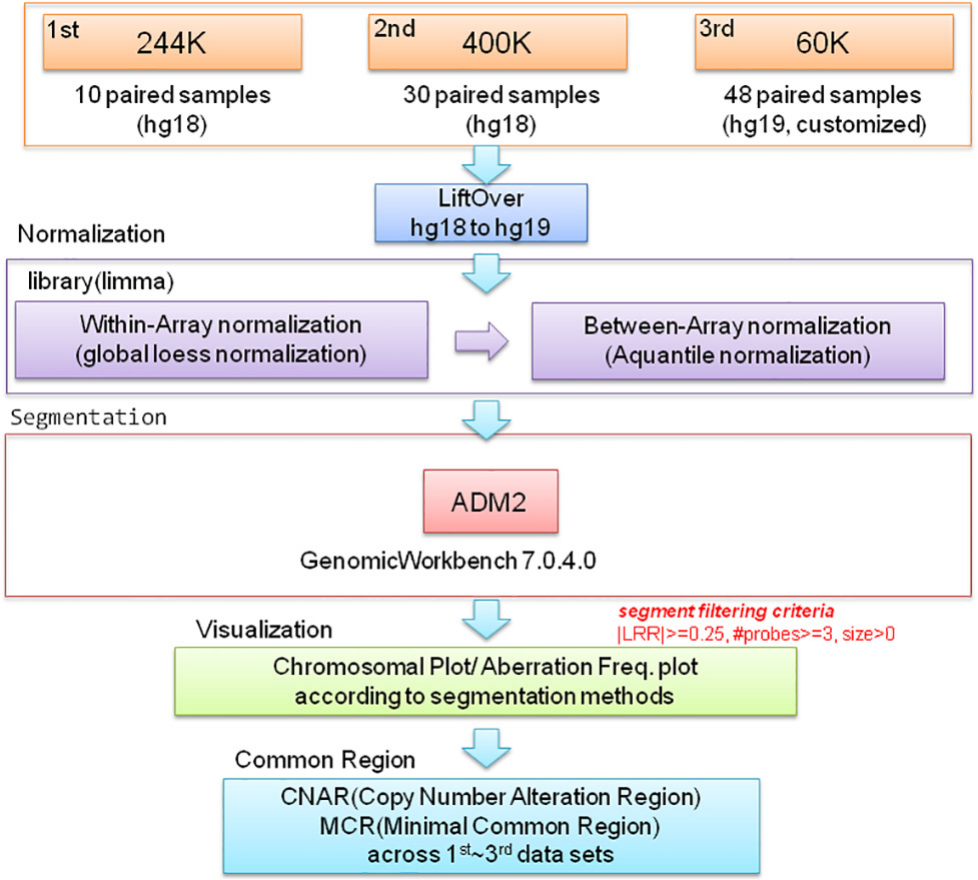
A schematic diagram for identifying a minimal common region. The Aberration Detection Method 2 (ADM-2) algorithm with a sensitivity threshold of 6.0 was used to identify the CNAs in gastric cancer and to determine the frequencies of CNAs in each sample. The MCR (Minimal Common Region) of copy number gains or losses was identified through the analysis of CNAs in the three kinds of aCGHs (244K, 400K, and 60K).

The aCGH was performed according to the manufacturer’s recommendations. After DNA hybridization and washing, slides were scanned immediately using an Agilent microarray scanner, and raw data were extracted using Feature Extraction Software at the default CGH parameter settings (Agilent Technologies). Putative CNA intervals in each sample were identified using Agilent Genomic Workbench v7.0.4.0 software. Cy5/Cy3 ratios were converted into log_2_-transformed values. Centralization and fuzzy zero corrections were applied to the microarray. The Aberration Detection Method 2 (ADM-2) algorithm at threshold 6.0 was used to identify the CNAs in individual samples and to determine aberration frequencies in gastric cancer samples (Fig 5). The following filters were employed: minimum number of probes in region > = 3, minimum absolute average log ratio of region > = 0.25. Common aberrations were detected by using the context-corrected algorithm at p-value < 0.05 and an overlap threshold of 0.9. The CNAR (Copy Number Alteration Region) was defined as the union of more than 90 percent overlapping aberrant segments across multiple samples. The UCSC genome assembly hg19 was used as the human reference genome sequence. For each platform (244K, 400K, and 60K), the within array global Lowess normalization method was applied to correct for local spatial bias and continuous spatial gradients. After the within array normalization, a quantile between array normalization was applied to compare the aberration results across arrays. These normalizations were carried out using the limma package in R. The MCR (Minimal Common Region) was defined as a 100 percent overlapping common region between samples in the CNAR. There are several MCRs in the CNAR according to the possible overlapping frequency. The MCR of amplification and deletion was analyzed. Amplification and deletion was defined when the normalized log2 ratio was ≥0.8 and ≤−0.8, respectively. All statistical methods and visualization of individual aberrant regions were conducted using R statistical language v.3.0.2 (www.r-project.org).

### Multiplex Ligation-Dependent Probe Amplification (MLPA) Analysis

MLPA analysis was performed using the SALSA MLPA kit P200 (MRC-Holland, Amsterdam, Netherlands) according to the manufacturer’s instructions [52]. The P200 kit contains 14 internal control probes to assess DNA denaturation and DNA quantity, and also for the X and Y chromosome. DNA samples were diluted with TE to 5 μl and were heated at 98°C for 5 min in PCR tubes in a thermocycler with a heated lid. After the addition of 1.5 μl MLPA buffer and 1.5 μl probe mix, samples were further heated for 1 min at 95°C and then incubated for 16 h at 60°C. The probe sequences for detected genes are listed in Table H in S1 File. Ligation of annealed oligonucleotides was performed by diluting the samples to 40 μl with a dilution buffer containing 1 U Ligase-65 enzyme, and incubating for 15 min at 54°C. The ligase enzyme was inactivated by heating at 98°C for 5 min and ligation products were amplified by PCR. While at 60°C, 10 μl of a buffered solution containing the PCR primers, dNTPs and SALSA polymerase (MRC-Holland, Amsterdam, Netherlands) were added. PCR was carried out for 35 cycles (30 s at 95°C, 30 s at 60°C and 1 min at 72°C). The MLPA PCR reactions were separated using the capillary electrophoresis system, ABI-Prism 3130 (Applied Biosystems, Foster City, CA), and the data was analyzed using a GeneMaker 2.0.0 (SoftGenetics, State College, PA). Data was population-normalized, and probe ratios below 0.75 were regarded as an indication of deletion, while probe ratios above 1.25 were regarded as an indication of amplification.

### Quantitative Real-Time PCR (qRT-PCR)

Total RNA was isolated using PureLink RNA Mini Kit (Invitrogen, Carlsbad, CA), and RT-PCR was carried out using SuperScript VILO cDNA Synthesis Kit (Invitrogen, Carlsbad, CA) according to the manufacturer's protocol. Real-time PCR was carried with SYBR green dye (Qiagen, Valencia, CA) under the following conditions: an initial denaturation step of 10 min at 95°C, followed by 40 cycles at 95°C for 15 s and 60°C for 30 s. The PCR primers (Table I in S1 File) were designed using Primer Express 3 (Applied Biosystems, Foster City, CA), and the specificity of primer sets was checked with BLAST. The target mRNA amount in each sample was normalized to an internal control of RPLP0, and fold change was calculated by comparing the tumor with its matched normal.

### Statistical analysis

Statistical significance of log2 ratio of mRNA fold change was analyzed by a one-sample t-test. Associations between CNAs of individual genes and Lauren’s classification were tested by the Pearson’s chi-square test (or Fisher’s exact test). Correlations between two continuous variables were analyzed using Spearman's (or Pearson’s) correlation coefficients. The agglomerative hierarchical clustering algorithm was used for detecting clusters in copy number alterations. The effect of CNAs on overall survival was analyzed by Kaplan-Meier survival curves, and the significance of differences in survival between the two groups was evaluated by the log-rank test. All statistical analyses were two-sided, with a 5% type I error rate.

## Supporting Information

S1 Fig The effect of *MYC* CNA on overall survival. The Kaplan-Meier approach was used to estimate survival curves according to the CNA of MYC. The effect of CNA of MYC on overall survival was analyzed using log-rank test in 88 gastric cancers (A), 43 diffuse type cancers (B), and 41 intestinal type cancers (C). The CNAs of MYC tended to reduce the overall survival rate in diffuse and intestinal types, but the difference was not statistically significant.
(TIF)

S1 File Titles of supporting tables. Table A. Minimal common regions of recurrent (>20%) copy number gains. Table B. Minimal common regions of recurrent (>20%) copy number losses. Table C. Copy number alterations according to Lauren’s classification. Table D. Prevalence of copy number gains at 8q24 and 20q11.21 (or 20113.12). Table E. Comparison of copy number losses between aCGH-244K/-400K and aCGH-60K. Table F. Minimal common regions of recurrent (>10%) amplifications or deletions. Table G. Comparison of recurrent amplifications or deletions among studies. Table H. MLPA probe sequences. Table I. qPCR primer sequences.
(PPTX)
