# Supplementary figures and images for: Copy Number Gains at 8q24 and 20q11-q13 in Gastric Cancer Are More Common in Intestinal-Type than Diffuse-Type
Source: PLoS One. 2015 Sep 11;10(9):e0137657. doi: 10.1371/journal.pone.0137657 (PMC4567330; doi:10.1371/journal.pone.0137657)

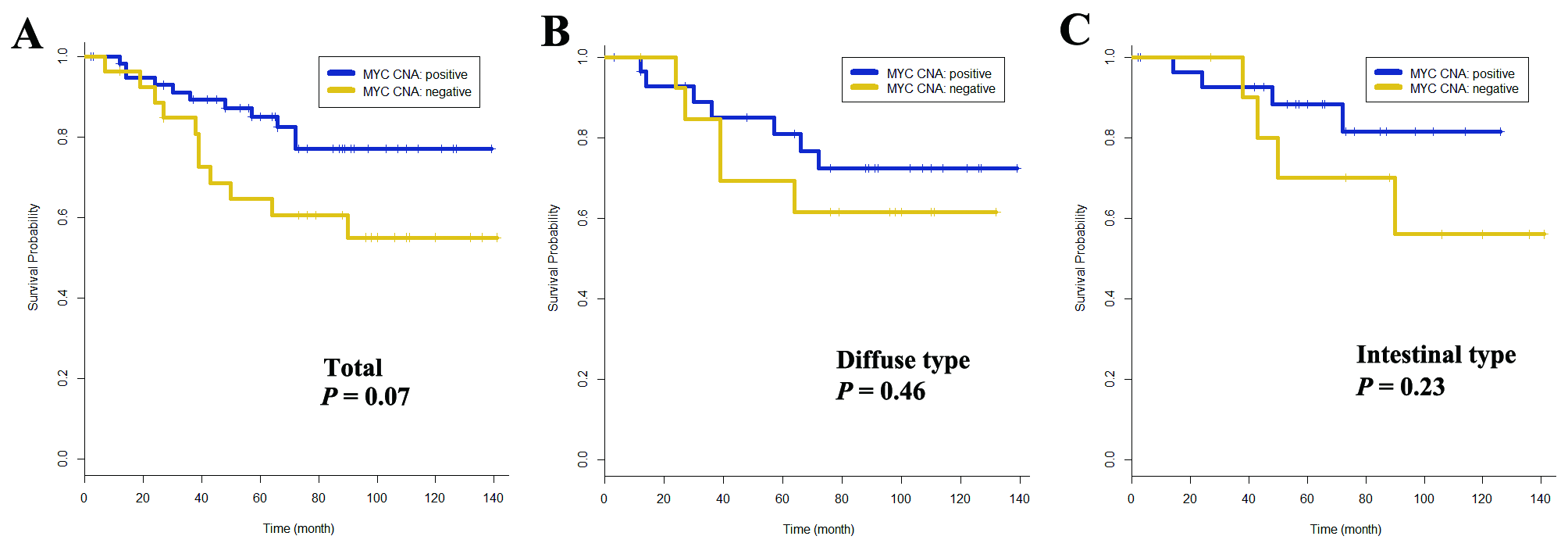

Supplement: S1 Fig — The Kaplan-Meier approach was used to estimate survival curves according to the CNA of MYC. The effect of CNA of MYC on overall survival was analyzed using log-rank test in 88 gastric cancers (A), 43 diffuse type cancers (B), and 41 intestinal type cancers (C). The CNAs of MYC tended to reduce the overall survival rate in diffuse and intestinal types, but the difference was not statistically significant. (TIF) [file pone.0137657.s001.tif]
