# Supplementary material for: Copy Number Gains at 8q24 and 20q11-q13 in Gastric Cancer Are More Common in Intestinal-Type than Diffuse-Type
Source: PLoS One. 2015 Sep 11;10(9):e0137657. doi: 10.1371/journal.pone.0137657 (PMC4567330; doi:10.1371/journal.pone.0137657)
Supplement: S1 File — Table A. Minimal common regions of recurrent (>20%) copy number gains. Table B. Minimal common regions of recurrent (>20%) copy number losses. Table C. Copy number alterations according to Lauren’s classification. Table D. Prevalence of copy number gains at 8q24 and 20q11.21 (or 20113.12). Table E. Comparison of copy number losses between aCGH-244K/-400K and aCGH-60K. Table F. Minimal common regions of recurrent (>10%) amplifications or deletions. Table G. Comparison of recurrent amplifications or deletions among studies. Table H. MLPA probe sequences. Table I. qPCR primer sequences. (PPTX) [file pone.0137657.s002.pptx]

## Slide 1
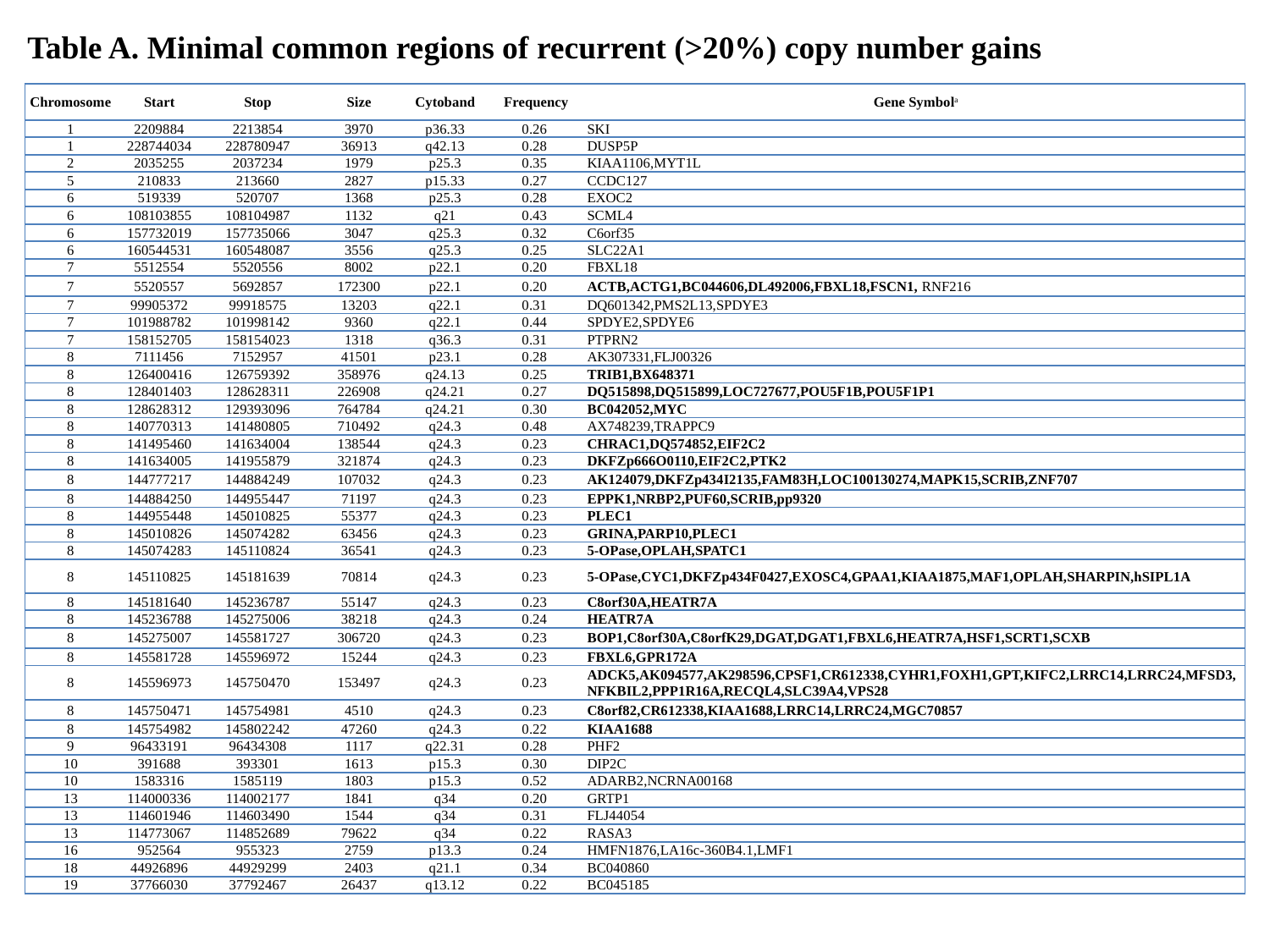

Table A. Minimal common regions of recurrent (>20%) copy number gains
| Chromosome | Start | Stop | Size | Cytoband | Frequency | Gene Symbola |
| --- | --- | --- | --- | --- | --- | --- |
| 1 | 2209884 | 2213854 | 3970 | p36.33 | 0.26 | SKI |
| 1 | 228744034 | 228780947 | 36913 | q42.13 | 0.28 | DUSP5P |
| 2 | 2035255 | 2037234 | 1979 | p25.3 | 0.35 | KIAA1106,MYT1L |
| 5 | 210833 | 213660 | 2827 | p15.33 | 0.27 | CCDC127 |
| 6 | 519339 | 520707 | 1368 | p25.3 | 0.28 | EXOC2 |
| 6 | 108103855 | 108104987 | 1132 | q21 | 0.43 | SCML4 |
| 6 | 157732019 | 157735066 | 3047 | q25.3 | 0.32 | C6orf35 |
| 6 | 160544531 | 160548087 | 3556 | q25.3 | 0.25 | SLC22A1 |
| 7 | 5512554 | 5520556 | 8002 | p22.1 | 0.20 | FBXL18 |
| 7 | 5520557 | 5692857 | 172300 | p22.1 | 0.20 | ACTB,ACTG1,BC044606,DL492006,FBXL18,FSCN1, RNF216 |
| 7 | 99905372 | 99918575 | 13203 | q22.1 | 0.31 | DQ601342,PMS2L13,SPDYE3 |
| 7 | 101988782 | 101998142 | 9360 | q22.1 | 0.44 | SPDYE2,SPDYE6 |
| 7 | 158152705 | 158154023 | 1318 | q36.3 | 0.31 | PTPRN2 |
| 8 | 7111456 | 7152957 | 41501 | p23.1 | 0.28 | AK307331,FLJ00326 |
| 8 | 126400416 | 126759392 | 358976 | q24.13 | 0.25 | TRIB1,BX648371 |
| 8 | 128401403 | 128628311 | 226908 | q24.21 | 0.27 | DQ515898,DQ515899,LOC727677,POU5F1B,POU5F1P1 |
| 8 | 128628312 | 129393096 | 764784 | q24.21 | 0.30 | BC042052,MYC |
| 8 | 140770313 | 141480805 | 710492 | q24.3 | 0.48 | AX748239,TRAPPC9 |
| 8 | 141495460 | 141634004 | 138544 | q24.3 | 0.23 | CHRAC1,DQ574852,EIF2C2 |
| 8 | 141634005 | 141955879 | 321874 | q24.3 | 0.23 | DKFZp666O0110,EIF2C2,PTK2 |
| 8 | 144777217 | 144884249 | 107032 | q24.3 | 0.23 | AK124079,DKFZp434I2135,FAM83H,LOC100130274,MAPK15,SCRIB,ZNF707 |
| 8 | 144884250 | 144955447 | 71197 | q24.3 | 0.23 | EPPK1,NRBP2,PUF60,SCRIB,pp9320 |
| 8 | 144955448 | 145010825 | 55377 | q24.3 | 0.23 | PLEC1 |
| 8 | 145010826 | 145074282 | 63456 | q24.3 | 0.23 | GRINA,PARP10,PLEC1 |
| 8 | 145074283 | 145110824 | 36541 | q24.3 | 0.23 | 5-OPase,OPLAH,SPATC1 |
| 8 | 145110825 | 145181639 | 70814 | q24.3 | 0.23 | 5-OPase,CYC1,DKFZp434F0427,EXOSC4,GPAA1,KIAA1875,MAF1,OPLAH,SHARPIN,hSIPL1A |
| 8 | 145181640 | 145236787 | 55147 | q24.3 | 0.23 | C8orf30A,HEATR7A |
| 8 | 145236788 | 145275006 | 38218 | q24.3 | 0.24 | HEATR7A |
| 8 | 145275007 | 145581727 | 306720 | q24.3 | 0.23 | BOP1,C8orf30A,C8orfK29,DGAT,DGAT1,FBXL6,HEATR7A,HSF1,SCRT1,SCXB |
| 8 | 145581728 | 145596972 | 15244 | q24.3 | 0.23 | FBXL6,GPR172A |
| 8 | 145596973 | 145750470 | 153497 | q24.3 | 0.23 | ADCK5,AK094577,AK298596,CPSF1,CR612338,CYHR1,FOXH1,GPT,KIFC2,LRRC14,LRRC24,MFSD3,NFKBIL2,PPP1R16A,RECQL4,SLC39A4,VPS28 |
| 8 | 145750471 | 145754981 | 4510 | q24.3 | 0.23 | C8orf82,CR612338,KIAA1688,LRRC14,LRRC24,MGC70857 |
| 8 | 145754982 | 145802242 | 47260 | q24.3 | 0.22 | KIAA1688 |
| 9 | 96433191 | 96434308 | 1117 | q22.31 | 0.28 | PHF2 |
| 10 | 391688 | 393301 | 1613 | p15.3 | 0.30 | DIP2C |
| 10 | 1583316 | 1585119 | 1803 | p15.3 | 0.52 | ADARB2,NCRNA00168 |
| 13 | 114000336 | 114002177 | 1841 | q34 | 0.20 | GRTP1 |
| 13 | 114601946 | 114603490 | 1544 | q34 | 0.31 | FLJ44054 |
| 13 | 114773067 | 114852689 | 79622 | q34 | 0.22 | RASA3 |
| 16 | 952564 | 955323 | 2759 | p13.3 | 0.24 | HMFN1876,LA16c-360B4.1,LMF1 |
| 18 | 44926896 | 44929299 | 2403 | q21.1 | 0.34 | BC040860 |
| 19 | 37766030 | 37792467 | 26437 | q13.12 | 0.22 | BC045185 |

## Slide 2
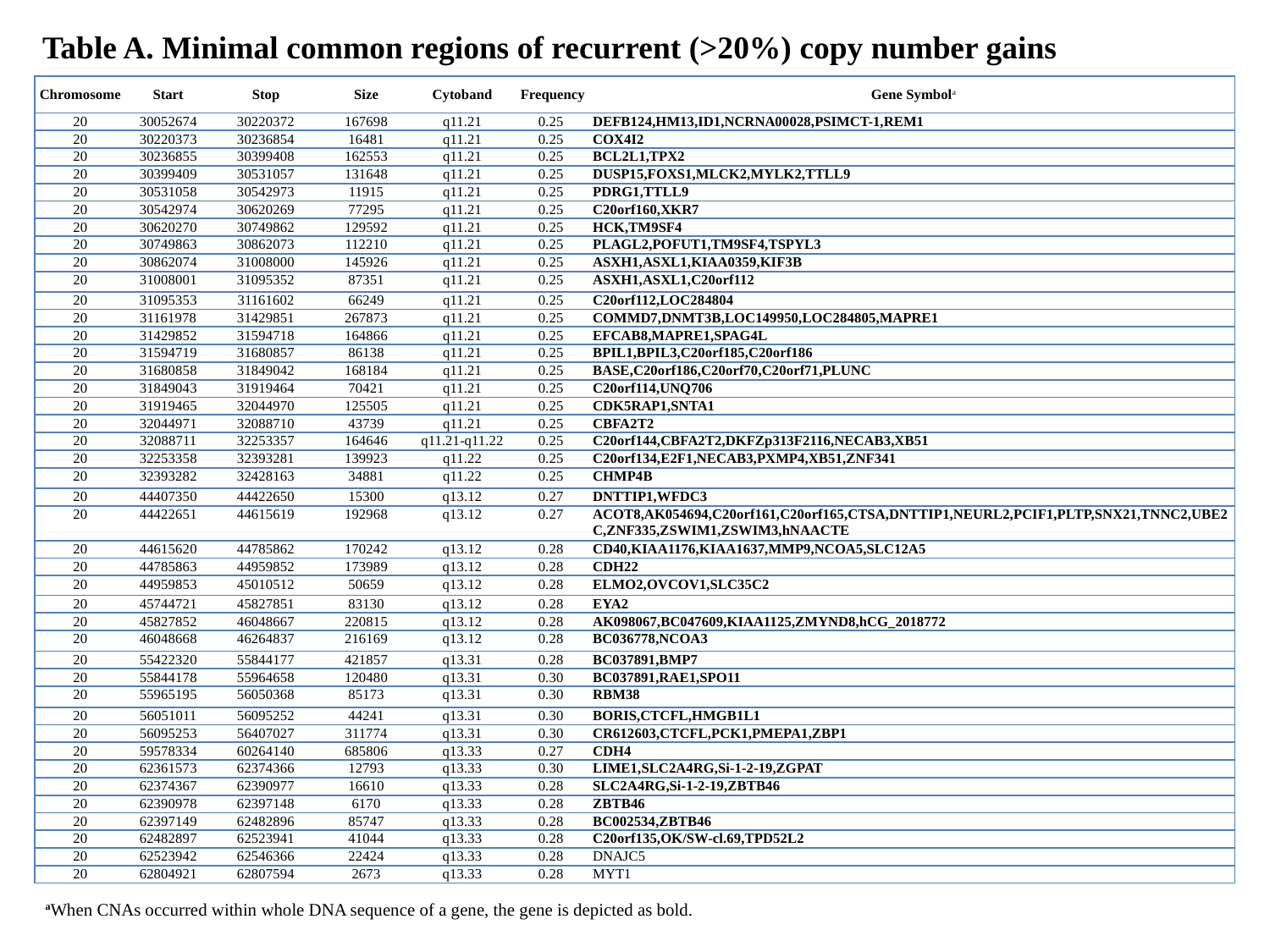

Table A. Minimal common regions of recurrent (>20%) copy number gains
| Chromosome | Start | Stop | Size | Cytoband | Frequency | Gene Symbola |
| --- | --- | --- | --- | --- | --- | --- |
| 20 | 30052674 | 30220372 | 167698 | q11.21 | 0.25 | DEFB124,HM13,ID1,NCRNA00028,PSIMCT-1,REM1 |
| 20 | 30220373 | 30236854 | 16481 | q11.21 | 0.25 | COX4I2 |
| 20 | 30236855 | 30399408 | 162553 | q11.21 | 0.25 | BCL2L1,TPX2 |
| 20 | 30399409 | 30531057 | 131648 | q11.21 | 0.25 | DUSP15,FOXS1,MLCK2,MYLK2,TTLL9 |
| 20 | 30531058 | 30542973 | 11915 | q11.21 | 0.25 | PDRG1,TTLL9 |
| 20 | 30542974 | 30620269 | 77295 | q11.21 | 0.25 | C20orf160,XKR7 |
| 20 | 30620270 | 30749862 | 129592 | q11.21 | 0.25 | HCK,TM9SF4 |
| 20 | 30749863 | 30862073 | 112210 | q11.21 | 0.25 | PLAGL2,POFUT1,TM9SF4,TSPYL3 |
| 20 | 30862074 | 31008000 | 145926 | q11.21 | 0.25 | ASXH1,ASXL1,KIAA0359,KIF3B |
| 20 | 31008001 | 31095352 | 87351 | q11.21 | 0.25 | ASXH1,ASXL1,C20orf112 |
| 20 | 31095353 | 31161602 | 66249 | q11.21 | 0.25 | C20orf112,LOC284804 |
| 20 | 31161978 | 31429851 | 267873 | q11.21 | 0.25 | COMMD7,DNMT3B,LOC149950,LOC284805,MAPRE1 |
| 20 | 31429852 | 31594718 | 164866 | q11.21 | 0.25 | EFCAB8,MAPRE1,SPAG4L |
| 20 | 31594719 | 31680857 | 86138 | q11.21 | 0.25 | BPIL1,BPIL3,C20orf185,C20orf186 |
| 20 | 31680858 | 31849042 | 168184 | q11.21 | 0.25 | BASE,C20orf186,C20orf70,C20orf71,PLUNC |
| 20 | 31849043 | 31919464 | 70421 | q11.21 | 0.25 | C20orf114,UNQ706 |
| 20 | 31919465 | 32044970 | 125505 | q11.21 | 0.25 | CDK5RAP1,SNTA1 |
| 20 | 32044971 | 32088710 | 43739 | q11.21 | 0.25 | CBFA2T2 |
| 20 | 32088711 | 32253357 | 164646 | q11.21-q11.22 | 0.25 | C20orf144,CBFA2T2,DKFZp313F2116,NECAB3,XB51 |
| 20 | 32253358 | 32393281 | 139923 | q11.22 | 0.25 | C20orf134,E2F1,NECAB3,PXMP4,XB51,ZNF341 |
| 20 | 32393282 | 32428163 | 34881 | q11.22 | 0.25 | CHMP4B |
| 20 | 44407350 | 44422650 | 15300 | q13.12 | 0.27 | DNTTIP1,WFDC3 |
| 20 | 44422651 | 44615619 | 192968 | q13.12 | 0.27 | ACOT8,AK054694,C20orf161,C20orf165,CTSA,DNTTIP1,NEURL2,PCIF1,PLTP,SNX21,TNNC2,UBE2C,ZNF335,ZSWIM1,ZSWIM3,hNAACTE |
| 20 | 44615620 | 44785862 | 170242 | q13.12 | 0.28 | CD40,KIAA1176,KIAA1637,MMP9,NCOA5,SLC12A5 |
| 20 | 44785863 | 44959852 | 173989 | q13.12 | 0.28 | CDH22 |
| 20 | 44959853 | 45010512 | 50659 | q13.12 | 0.28 | ELMO2,OVCOV1,SLC35C2 |
| 20 | 45744721 | 45827851 | 83130 | q13.12 | 0.28 | EYA2 |
| 20 | 45827852 | 46048667 | 220815 | q13.12 | 0.28 | AK098067,BC047609,KIAA1125,ZMYND8,hCG\_2018772 |
| 20 | 46048668 | 46264837 | 216169 | q13.12 | 0.28 | BC036778,NCOA3 |
| 20 | 55422320 | 55844177 | 421857 | q13.31 | 0.28 | BC037891,BMP7 |
| 20 | 55844178 | 55964658 | 120480 | q13.31 | 0.30 | BC037891,RAE1,SPO11 |
| 20 | 55965195 | 56050368 | 85173 | q13.31 | 0.30 | RBM38 |
| 20 | 56051011 | 56095252 | 44241 | q13.31 | 0.30 | BORIS,CTCFL,HMGB1L1 |
| 20 | 56095253 | 56407027 | 311774 | q13.31 | 0.30 | CR612603,CTCFL,PCK1,PMEPA1,ZBP1 |
| 20 | 59578334 | 60264140 | 685806 | q13.33 | 0.27 | CDH4 |
| 20 | 62361573 | 62374366 | 12793 | q13.33 | 0.30 | LIME1,SLC2A4RG,Si-1-2-19,ZGPAT |
| 20 | 62374367 | 62390977 | 16610 | q13.33 | 0.28 | SLC2A4RG,Si-1-2-19,ZBTB46 |
| 20 | 62390978 | 62397148 | 6170 | q13.33 | 0.28 | ZBTB46 |
| 20 | 62397149 | 62482896 | 85747 | q13.33 | 0.28 | BC002534,ZBTB46 |
| 20 | 62482897 | 62523941 | 41044 | q13.33 | 0.28 | C20orf135,OK/SW-cl.69,TPD52L2 |
| 20 | 62523942 | 62546366 | 22424 | q13.33 | 0.28 | DNAJC5 |
| 20 | 62804921 | 62807594 | 2673 | q13.33 | 0.28 | MYT1 |
 aWhen CNAs occurred within whole DNA sequence of a gene, the gene is depicted as bold.

## Slide 3
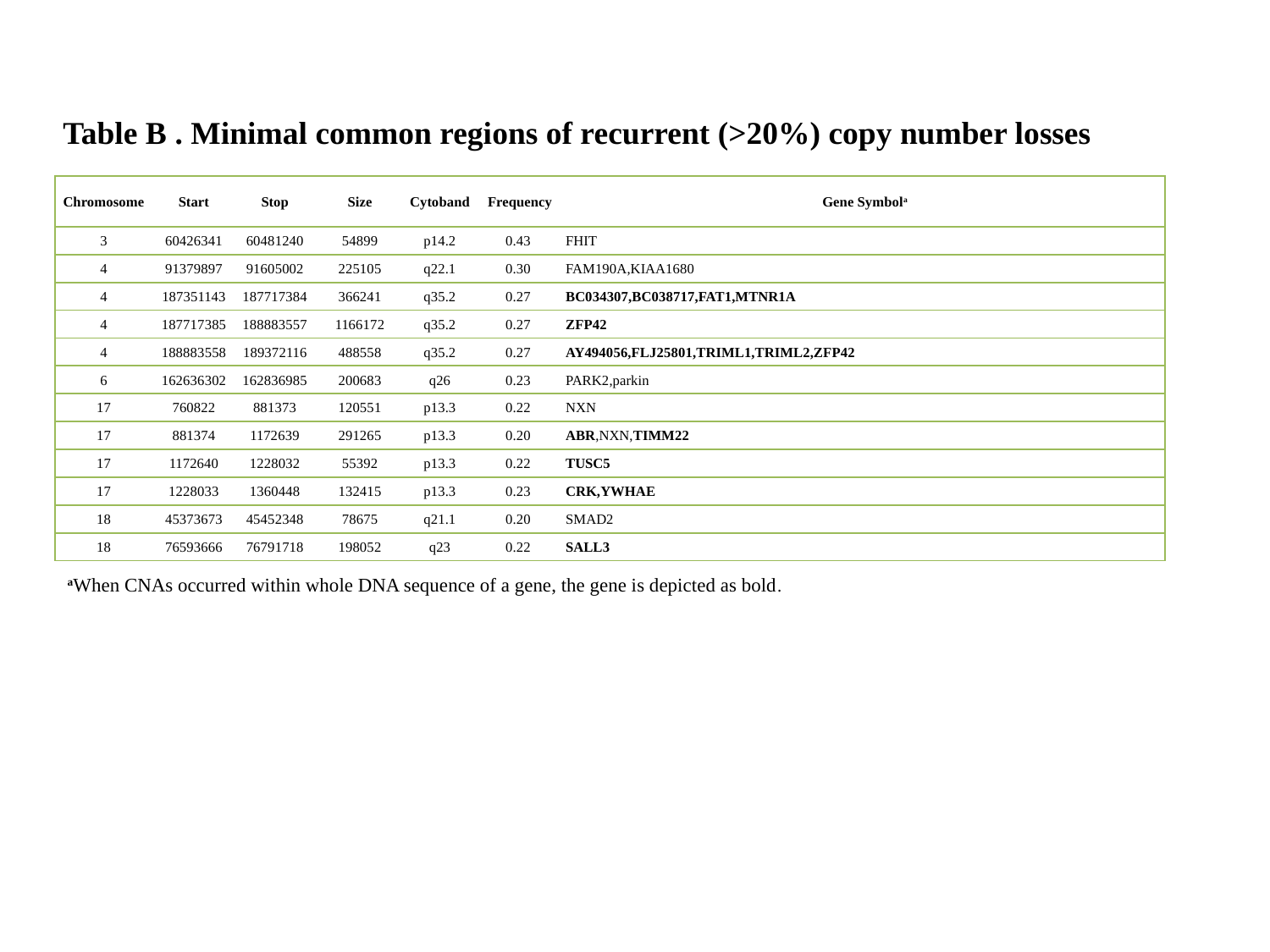

Table B . Minimal common regions of recurrent (>20%) copy number losses
| Chromosome | Start | Stop | Size | Cytoband | Frequency | Gene Symbola |
| --- | --- | --- | --- | --- | --- | --- |
| 3 | 60426341 | 60481240 | 54899 | p14.2 | 0.43 | FHIT |
| 4 | 91379897 | 91605002 | 225105 | q22.1 | 0.30 | FAM190A,KIAA1680 |
| 4 | 187351143 | 187717384 | 366241 | q35.2 | 0.27 | BC034307,BC038717,FAT1,MTNR1A |
| 4 | 187717385 | 188883557 | 1166172 | q35.2 | 0.27 | ZFP42 |
| 4 | 188883558 | 189372116 | 488558 | q35.2 | 0.27 | AY494056,FLJ25801,TRIML1,TRIML2,ZFP42 |
| 6 | 162636302 | 162836985 | 200683 | q26 | 0.23 | PARK2,parkin |
| 17 | 760822 | 881373 | 120551 | p13.3 | 0.22 | NXN |
| 17 | 881374 | 1172639 | 291265 | p13.3 | 0.20 | ABR,NXN,TIMM22 |
| 17 | 1172640 | 1228032 | 55392 | p13.3 | 0.22 | TUSC5 |
| 17 | 1228033 | 1360448 | 132415 | p13.3 | 0.23 | CRK,YWHAE |
| 18 | 45373673 | 45452348 | 78675 | q21.1 | 0.20 | SMAD2 |
| 18 | 76593666 | 76791718 | 198052 | q23 | 0.22 | SALL3 |
aWhen CNAs occurred within whole DNA sequence of a gene, the gene is depicted as bold.

## Slide 4
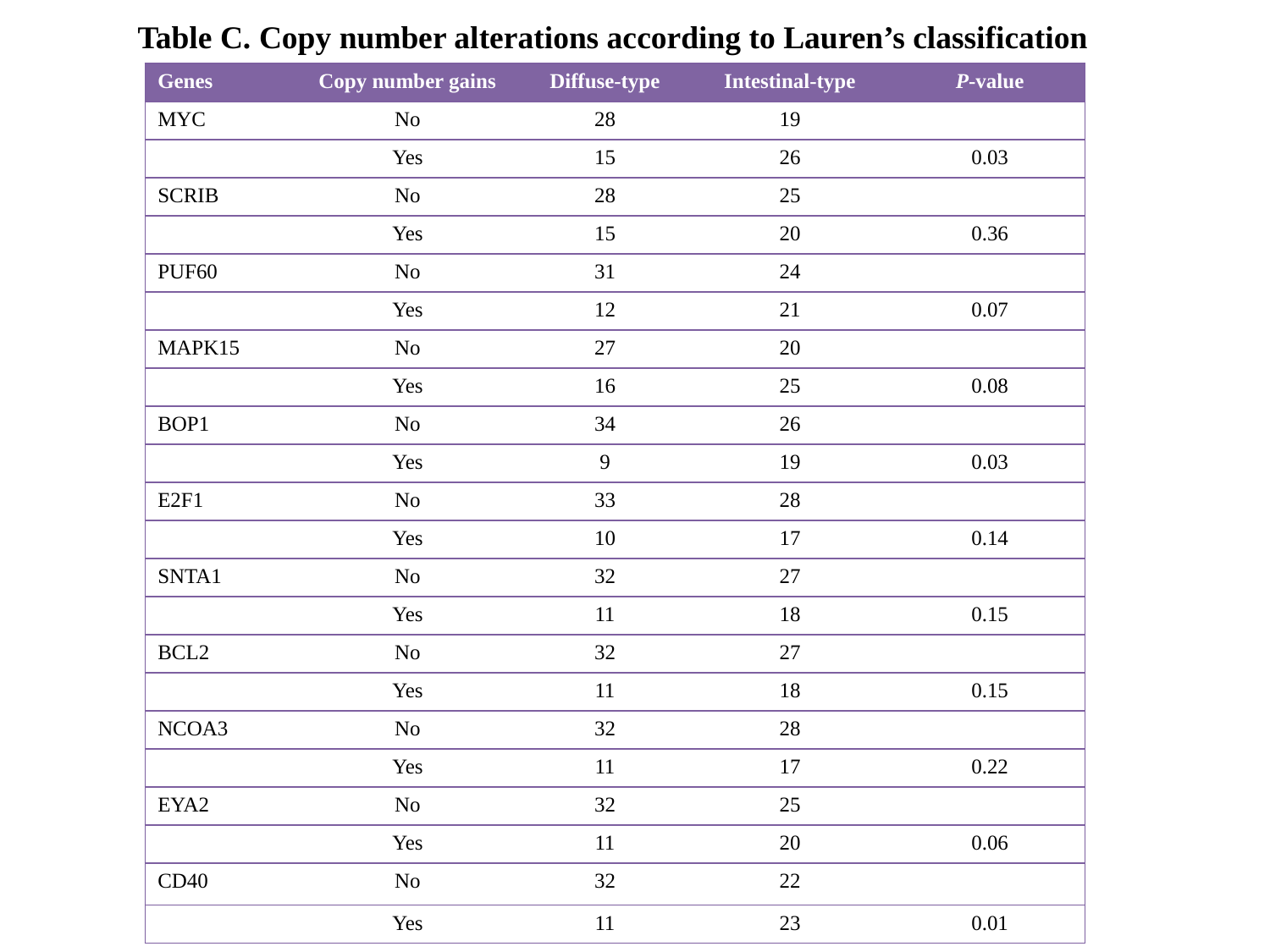

Table C. Copy number alterations according to Lauren’s classification
| Genes | Copy number gains | Diffuse-type | Intestinal-type | P-value |
| --- | --- | --- | --- | --- |
| MYC | No | 28 | 19 | |
| | Yes | 15 | 26 | 0.03 |
| SCRIB | No | 28 | 25 | |
| | Yes | 15 | 20 | 0.36 |
| PUF60 | No | 31 | 24 | |
| | Yes | 12 | 21 | 0.07 |
| MAPK15 | No | 27 | 20 | |
| | Yes | 16 | 25 | 0.08 |
| BOP1 | No | 34 | 26 | |
| | Yes | 9 | 19 | 0.03 |
| E2F1 | No | 33 | 28 | |
| | Yes | 10 | 17 | 0.14 |
| SNTA1 | No | 32 | 27 | |
| | Yes | 11 | 18 | 0.15 |
| BCL2 | No | 32 | 27 | |
| | Yes | 11 | 18 | 0.15 |
| NCOA3 | No | 32 | 28 | |
| | Yes | 11 | 17 | 0.22 |
| EYA2 | No | 32 | 25 | |
| | Yes | 11 | 20 | 0.06 |
| CD40 | No | 32 | 22 | |
| | Yes | 11 | 23 | 0.01 |

## Slide 5
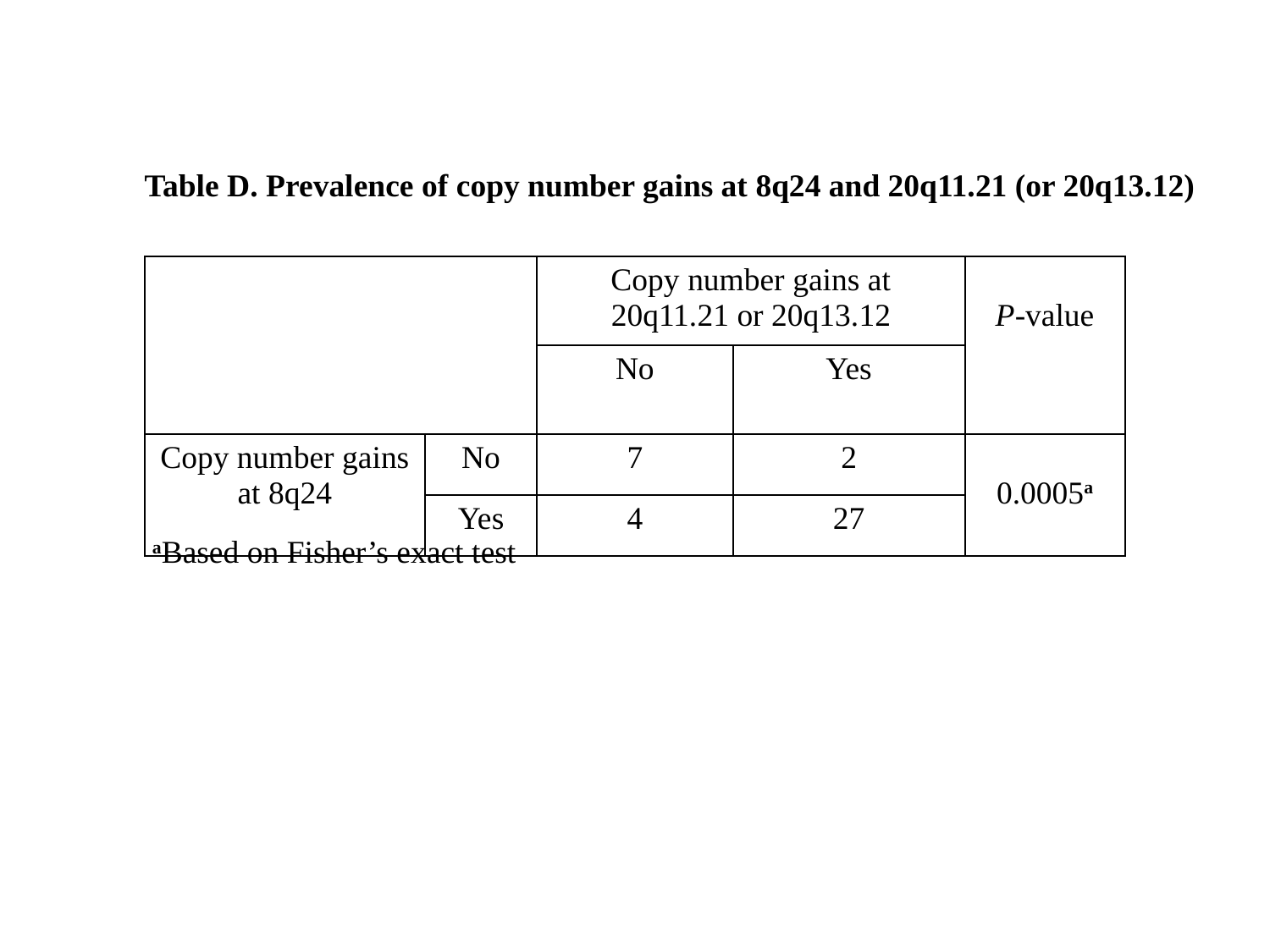

Table D. Prevalence of copy number gains at 8q24 and 20q11.21 (or 20q13.12)
| | | Copy number gains at 20q11.21 or 20q13.12 | | P-value |
| --- | --- | --- | --- | --- |
| | | No | Yes | |
| Copy number gains at 8q24 | No | 7 | 2 | 0.0005a |
| | Yes | 4 | 27 | |
aBased on Fisher’s exact test

## Slide 6
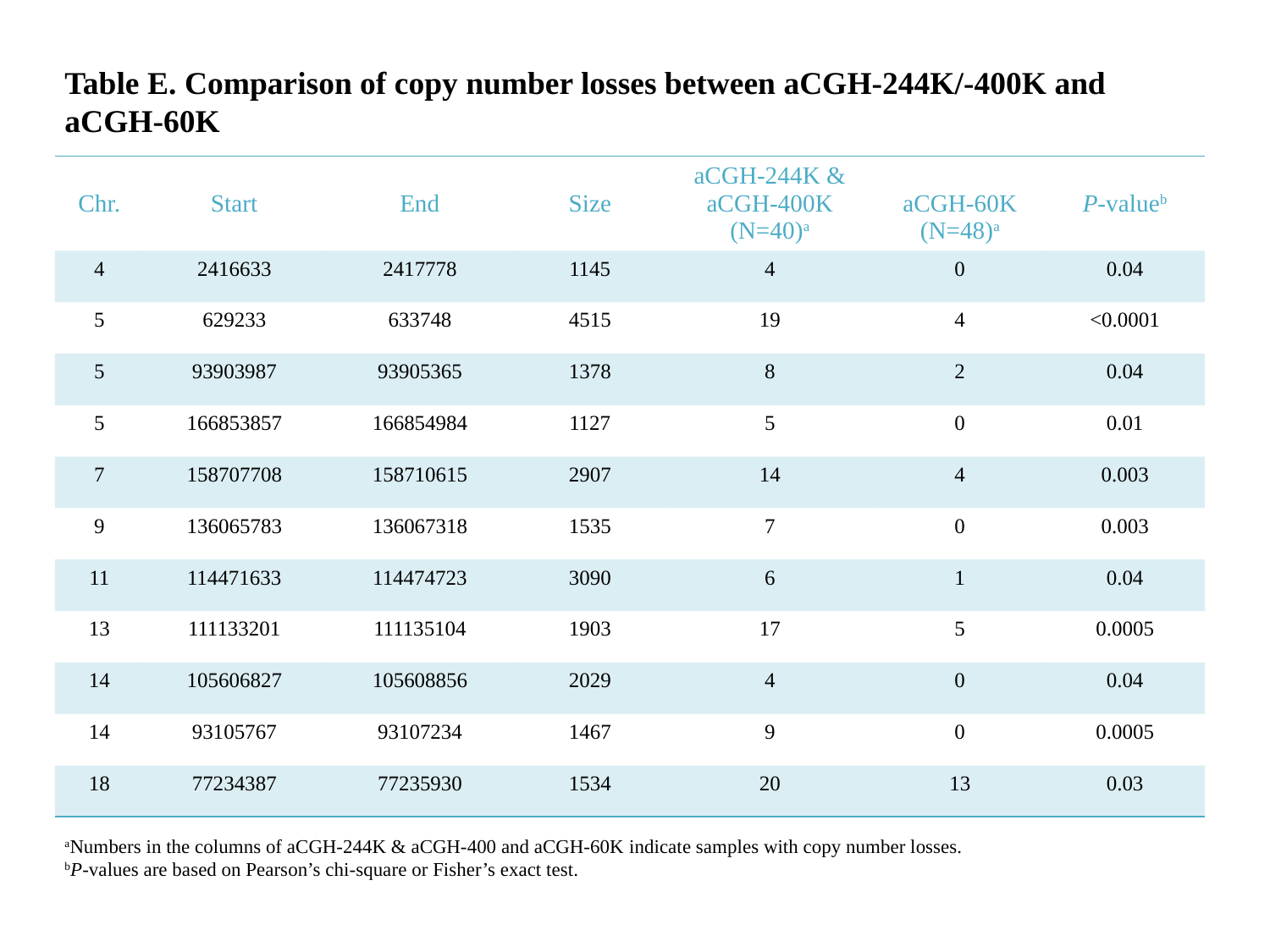

Table E. Comparison of copy number losses between aCGH-244K/-400K and
aCGH-60K
| Chr. | Start | End | Size | aCGH-244K & aCGH-400K (N=40)a | aCGH-60K (N=48)a | P-valueb |
| --- | --- | --- | --- | --- | --- | --- |
| 4 | 2416633 | 2417778 | 1145 | 4 | 0 | 0.04 |
| 5 | 629233 | 633748 | 4515 | 19 | 4 | <0.0001 |
| 5 | 93903987 | 93905365 | 1378 | 8 | 2 | 0.04 |
| 5 | 166853857 | 166854984 | 1127 | 5 | 0 | 0.01 |
| 7 | 158707708 | 158710615 | 2907 | 14 | 4 | 0.003 |
| 9 | 136065783 | 136067318 | 1535 | 7 | 0 | 0.003 |
| 11 | 114471633 | 114474723 | 3090 | 6 | 1 | 0.04 |
| 13 | 111133201 | 111135104 | 1903 | 17 | 5 | 0.0005 |
| 14 | 105606827 | 105608856 | 2029 | 4 | 0 | 0.04 |
| 14 | 93105767 | 93107234 | 1467 | 9 | 0 | 0.0005 |
| 18 | 77234387 | 77235930 | 1534 | 20 | 13 | 0.03 |
aNumbers in the columns of aCGH-244K & aCGH-400 and aCGH-60K indicate samples with copy number losses.
bP-values are based on Pearson’s chi-square or Fisher’s exact test.

## Slide 7
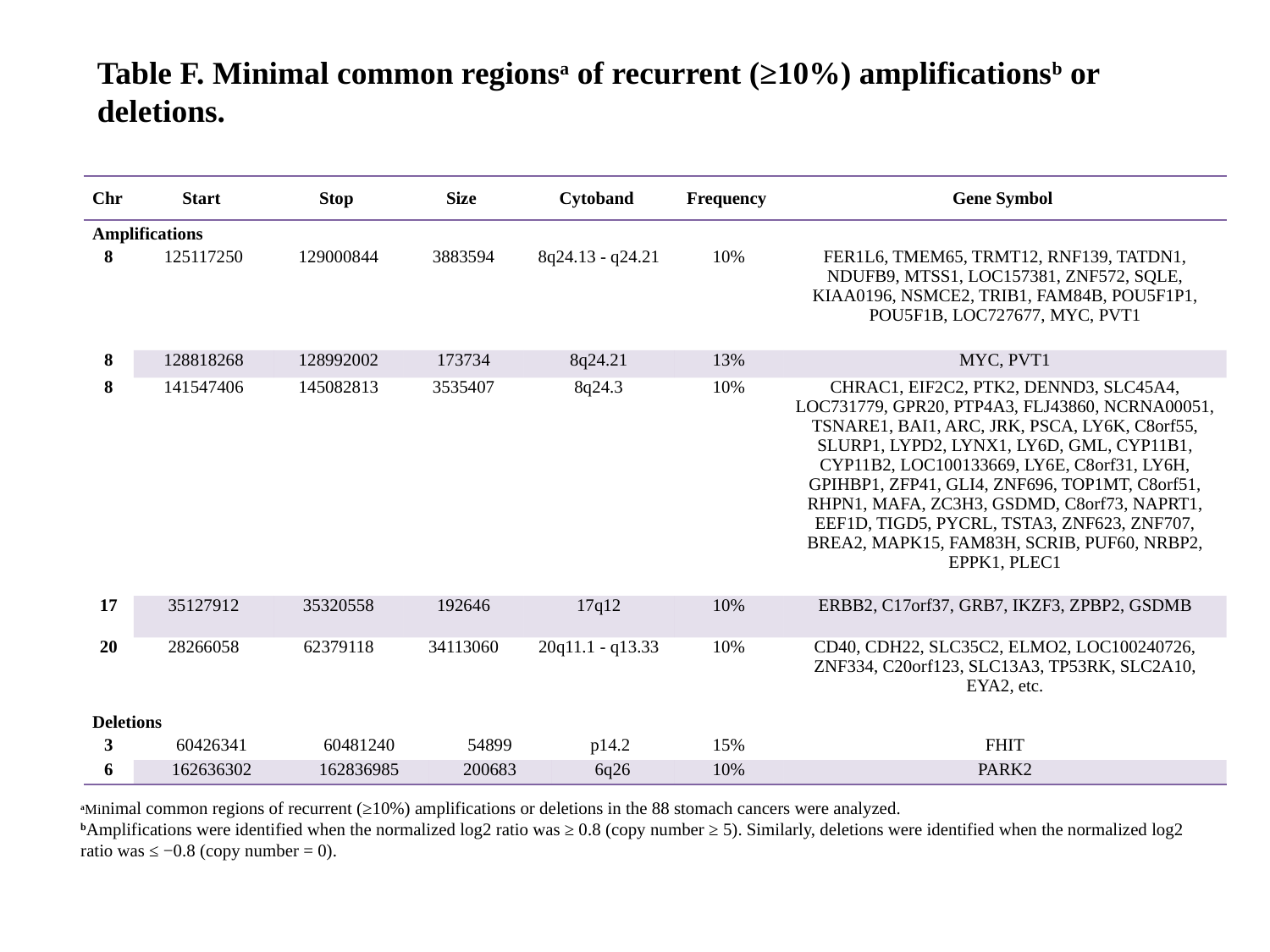

Table F. Minimal common regionsa of recurrent (≥10%) amplificationsb or deletions.
| Chr | Start | Stop | | Size | | Cytoband | | Frequency | Gene Symbol |
| --- | --- | --- | --- | --- | --- | --- | --- | --- | --- |
| Amplifications | | | | | | | | | |
| 8 | 125117250 | 129000844 | | 3883594 | | 8q24.13 - q24.21 | | 10% | FER1L6, TMEM65, TRMT12, RNF139, TATDN1, NDUFB9, MTSS1, LOC157381, ZNF572, SQLE, KIAA0196, NSMCE2, TRIB1, FAM84B, POU5F1P1, POU5F1B, LOC727677, MYC, PVT1 |
| 8 | 128818268 | 128992002 | | 173734 | | 8q24.21 | | 13% | MYC, PVT1 |
| 8 | 141547406 | 145082813 | | 3535407 | | 8q24.3 | | 10% | CHRAC1, EIF2C2, PTK2, DENND3, SLC45A4, LOC731779, GPR20, PTP4A3, FLJ43860, NCRNA00051, TSNARE1, BAI1, ARC, JRK, PSCA, LY6K, C8orf55, SLURP1, LYPD2, LYNX1, LY6D, GML, CYP11B1, CYP11B2, LOC100133669, LY6E, C8orf31, LY6H, GPIHBP1, ZFP41, GLI4, ZNF696, TOP1MT, C8orf51, RHPN1, MAFA, ZC3H3, GSDMD, C8orf73, NAPRT1, EEF1D, TIGD5, PYCRL, TSTA3, ZNF623, ZNF707, BREA2, MAPK15, FAM83H, SCRIB, PUF60, NRBP2, EPPK1, PLEC1 |
| 17 | 35127912 | 35320558 | | 192646 | | 17q12 | | 10% | ERBB2, C17orf37, GRB7, IKZF3, ZPBP2, GSDMB |
| 20 | 28266058 | 62379118 | | 34113060 | | 20q11.1 - q13.33 | | 10% | CD40, CDH22, SLC35C2, ELMO2, LOC100240726, ZNF334, C20orf123, SLC13A3, TP53RK, SLC2A10, EYA2, etc. |
| Deletions | | | | | | | | | |
| 3 | 60426341 | | 60481240 | | 54899 | | p14.2 | 15% | FHIT |
| 6 | 162636302 | | 162836985 | | 200683 | | 6q26 | 10% | PARK2 |
aMinimal common regions of recurrent (≥10%) amplifications or deletions in the 88 stomach cancers were analyzed.
bAmplifications were identified when the normalized log2 ratio was ≥ 0.8 (copy number ≥ 5). Similarly, deletions were identified when the normalized log2 ratio was ≤ −0.8 (copy number = 0).

## Slide 8
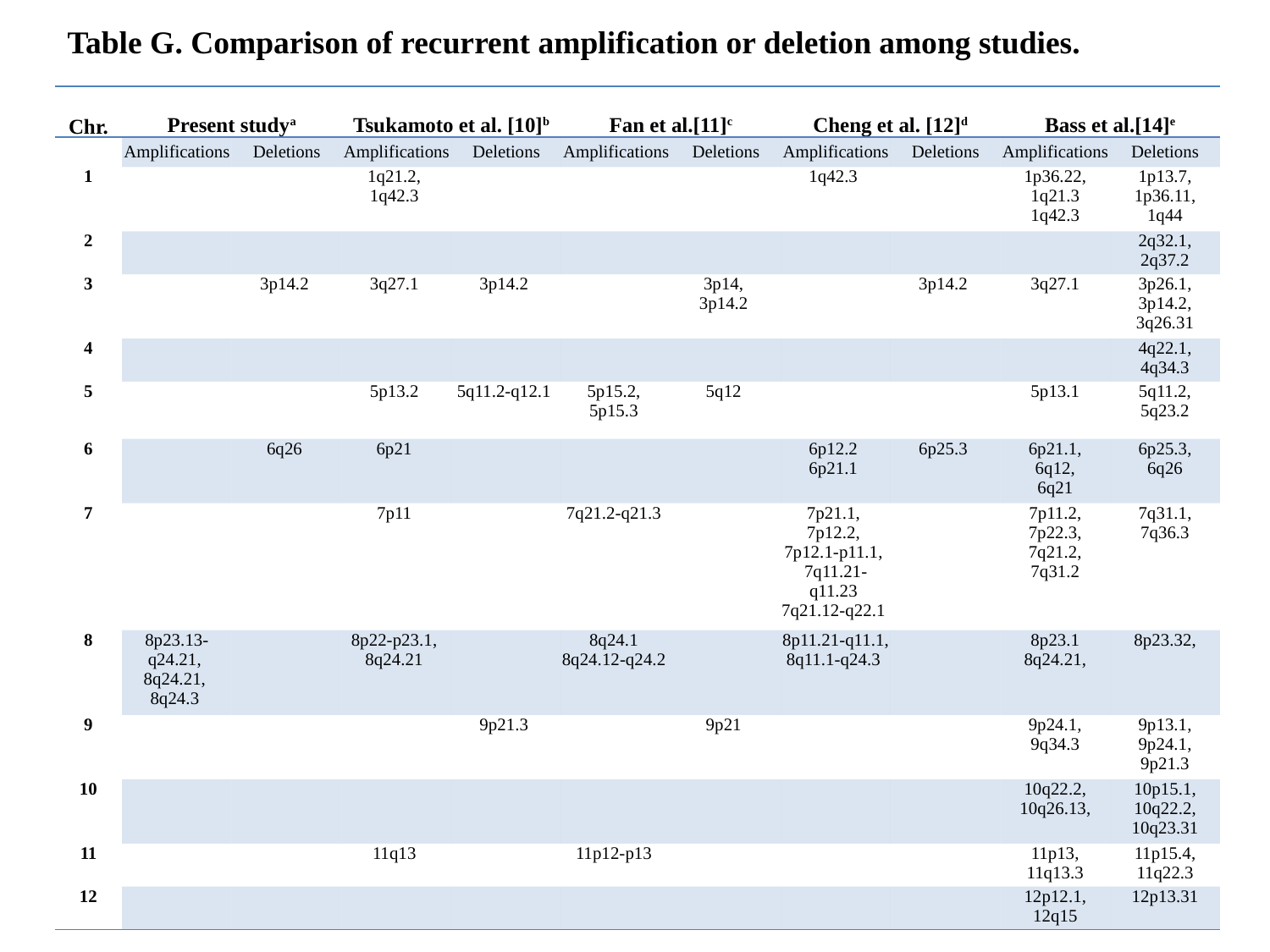

Table G. Comparison of recurrent amplification or deletion among studies.
| Chr. | Present studya | | Tsukamoto et al. [10]b | | Fan et al.[11]c | | Cheng et al. [12]d | | Bass et al.[14]e | |
| --- | --- | --- | --- | --- | --- | --- | --- | --- | --- | --- |
| | Amplifications | Deletions | Amplifications | Deletions | Amplifications | Deletions | Amplifications | Deletions | Amplifications | Deletions |
| 1 | | | 1q21.2, 1q42.3 | | | | 1q42.3 | | 1p36.22, 1q21.3 1q42.3 | 1p13.7, 1p36.11, 1q44 |
| 2 | | | | | | | | | | 2q32.1, 2q37.2 |
| 3 | | 3p14.2 | 3q27.1 | 3p14.2 | | 3p14, 3p14.2 | | 3p14.2 | 3q27.1 | 3p26.1, 3p14.2, 3q26.31 |
| 4 | | | | | | | | | | 4q22.1, 4q34.3 |
| 5 | | | 5p13.2 | 5q11.2-q12.1 | 5p15.2, 5p15.3 | 5q12 | | | 5p13.1 | 5q11.2, 5q23.2 |
| 6 | | 6q26 | 6p21 | | | | 6p12.2 6p21.1 | 6p25.3 | 6p21.1, 6q12, 6q21 | 6p25.3, 6q26 |
| 7 | | | 7p11 | | 7q21.2-q21.3 | | 7p21.1, 7p12.2, 7p12.1-p11.1, 7q11.21-q11.23 7q21.12-q22.1 | | 7p11.2, 7p22.3, 7q21.2, 7q31.2 | 7q31.1, 7q36.3 |
| 8 | 8p23.13-q24.21, 8q24.21, 8q24.3 | | 8p22-p23.1, 8q24.21 | | 8q24.1 8q24.12-q24.2 | | 8p11.21-q11.1, 8q11.1-q24.3 | | 8p23.1 8q24.21, | 8p23.32, |
| 9 | | | | 9p21.3 | | 9p21 | | | 9p24.1, 9q34.3 | 9p13.1, 9p24.1, 9p21.3 |
| 10 | | | | | | | | | 10q22.2, 10q26.13, | 10p15.1, 10q22.2, 10q23.31 |
| 11 | | | 11q13 | | 11p12-p13 | | | | 11p13, 11q13.3 | 11p15.4, 11q22.3 |
| 12 | | | | | | | | | 12p12.1, 12q15 | 12p13.31 |

## Slide 9
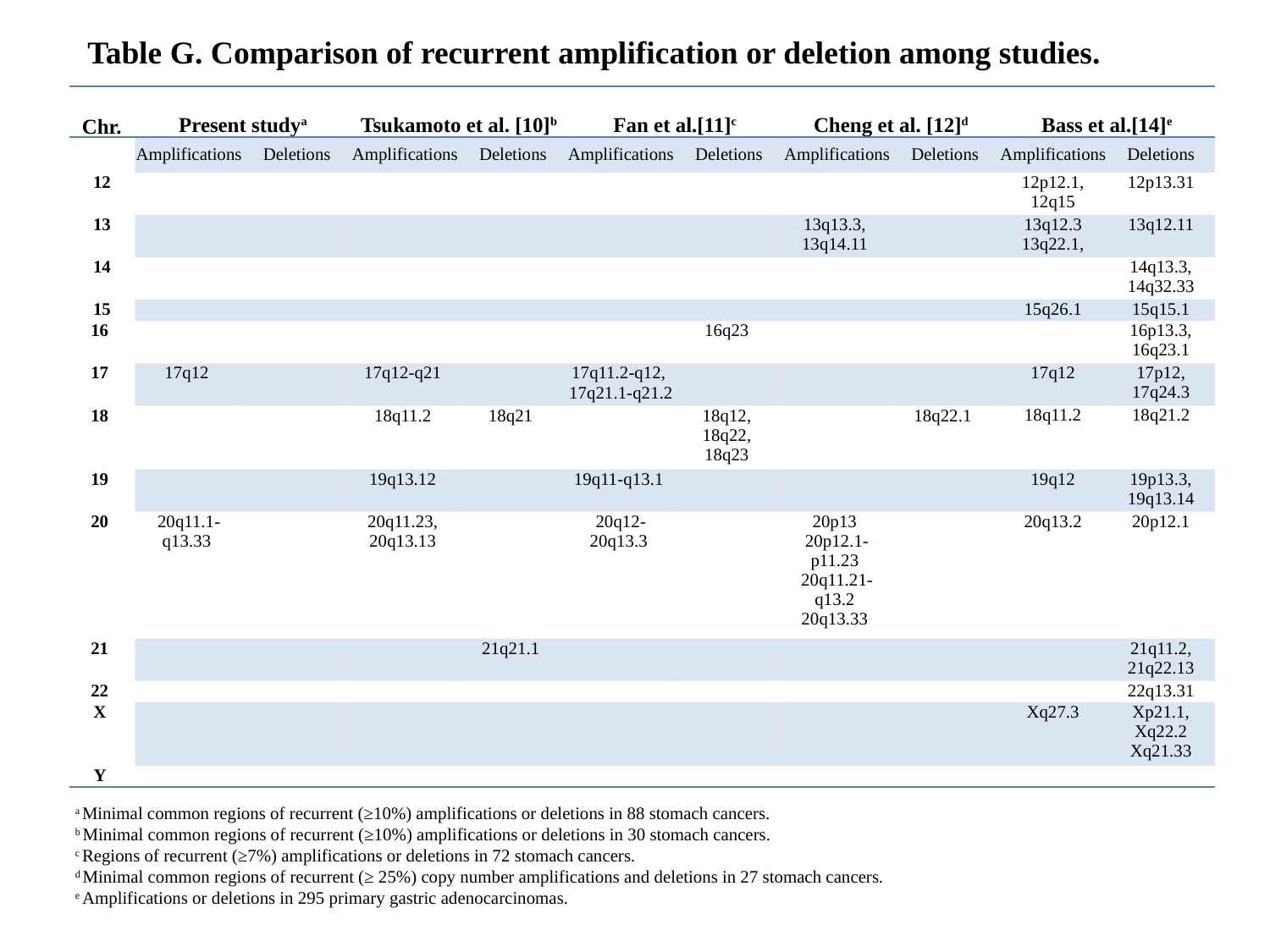

Table G. Comparison of recurrent amplification or deletion among studies.
| Chr. | Present studya | | Tsukamoto et al. [10]b | | Fan et al.[11]c | | Cheng et al. [12]d | | Bass et al.[14]e | |
| --- | --- | --- | --- | --- | --- | --- | --- | --- | --- | --- |
| | Amplifications | Deletions | Amplifications | Deletions | Amplifications | Deletions | Amplifications | Deletions | Amplifications | Deletions |
| 12 | | | | | | | | | 12p12.1, 12q15 | 12p13.31 |
| 13 | | | | | | | 13q13.3, 13q14.11 | | 13q12.3 13q22.1, | 13q12.11 |
| 14 | | | | | | | | | | 14q13.3, 14q32.33 |
| 15 | | | | | | | | | 15q26.1 | 15q15.1 |
| 16 | | | | | | 16q23 | | | | 16p13.3, 16q23.1 |
| 17 | 17q12 | | 17q12-q21 | | 17q11.2-q12, 17q21.1-q21.2 | | | | 17q12 | 17p12, 17q24.3 |
| 18 | | | 18q11.2 | 18q21 | | 18q12, 18q22, 18q23 | | 18q22.1 | 18q11.2 | 18q21.2 |
| 19 | | | 19q13.12 | | 19q11-q13.1 | | | | 19q12 | 19p13.3, 19q13.14 |
| 20 | 20q11.1-q13.33 | | 20q11.23, 20q13.13 | | 20q12-20q13.3 | | 20p13 20p12.1-p11.23 20q11.21-q13.2 20q13.33 | | 20q13.2 | 20p12.1 |
| 21 | | | | 21q21.1 | | | | | | 21q11.2, 21q22.13 |
| 22 | | | | | | | | | | 22q13.31 |
| X | | | | | | | | | Xq27.3 | Xp21.1, Xq22.2 Xq21.33 |
| Y | | | | | | | | | | |
a Minimal common regions of recurrent (≥10%) amplifications or deletions in 88 stomach cancers.
b Minimal common regions of recurrent (≥10%) amplifications or deletions in 30 stomach cancers.
c Regions of recurrent (≥7%) amplifications or deletions in 72 stomach cancers.
d Minimal common regions of recurrent (≥ 25%) copy number amplifications and deletions in 27 stomach cancers.
e Amplifications or deletions in 295 primary gastric adenocarcinomas.

## Slide 10
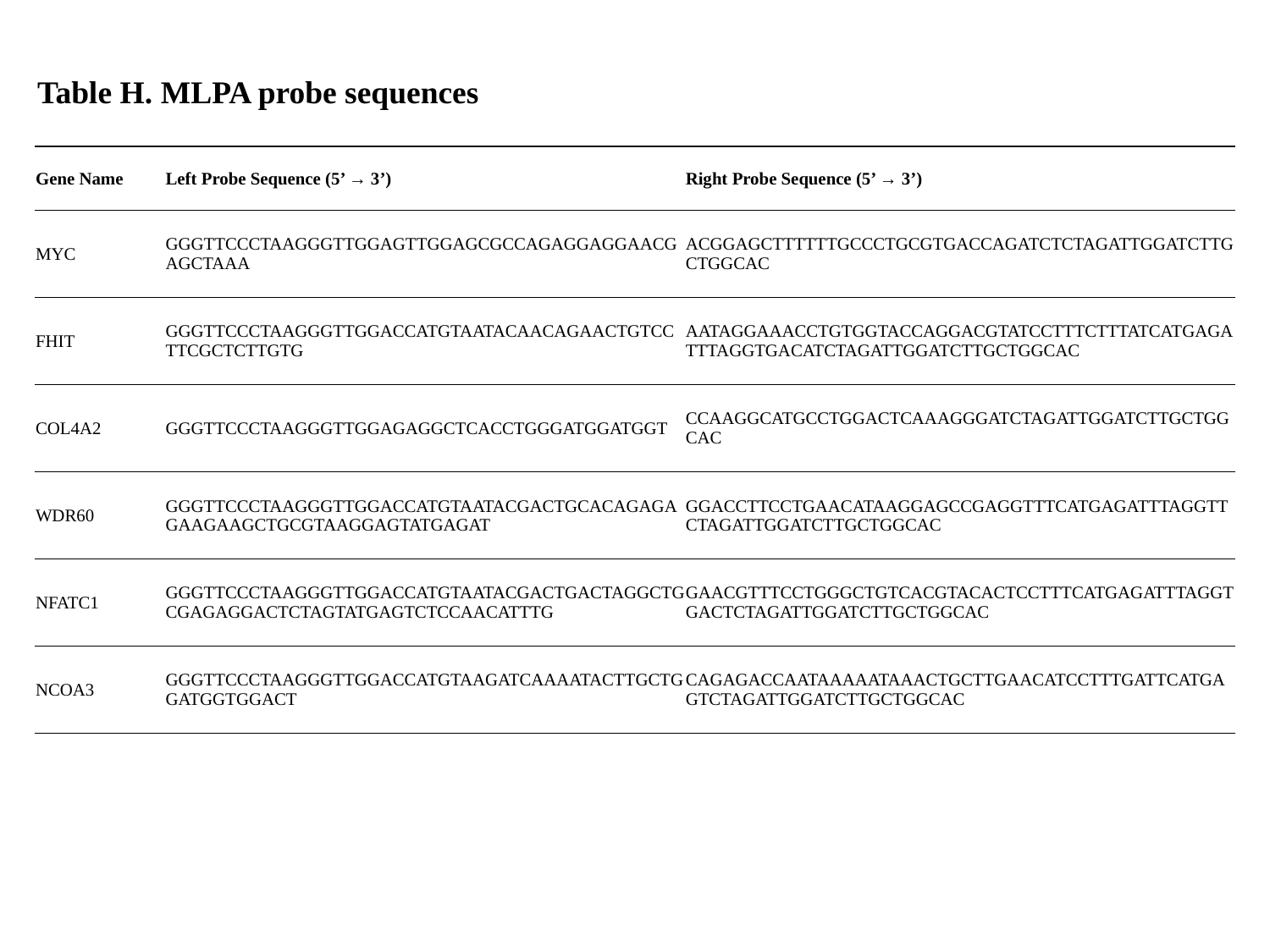

Table H. MLPA probe sequences
| Gene Name | Left Probe Sequence (5’ → 3’) | Right Probe Sequence (5’ → 3’) |
| --- | --- | --- |
| MYC | GGGTTCCCTAAGGGTTGGAGTTGGAGCGCCAGAGGAGGAACGAGCTAAA | ACGGAGCTTTTTTGCCCTGCGTGACCAGATCTCTAGATTGGATCTTGCTGGCAC |
| FHIT | GGGTTCCCTAAGGGTTGGACCATGTAATACAACAGAACTGTCCTTCGCTCTTGTG | AATAGGAAACCTGTGGTACCAGGACGTATCCTTTCTTTATCATGAGATTTAGGTGACATCTAGATTGGATCTTGCTGGCAC |
| COL4A2 | GGGTTCCCTAAGGGTTGGAGAGGCTCACCTGGGATGGATGGT | CCAAGGCATGCCTGGACTCAAAGGGATCTAGATTGGATCTTGCTGGCAC |
| WDR60 | GGGTTCCCTAAGGGTTGGACCATGTAATACGACTGCACAGAGAGAAGAAGCTGCGTAAGGAGTATGAGAT | GGACCTTCCTGAACATAAGGAGCCGAGGTTTCATGAGATTTAGGTTCTAGATTGGATCTTGCTGGCAC |
| NFATC1 | GGGTTCCCTAAGGGTTGGACCATGTAATACGACTGACTAGGCTGCGAGAGGACTCTAGTATGAGTCTCCAACATTTG | GAACGTTTCCTGGGCTGTCACGTACACTCCTTTCATGAGATTTAGGTGACTCTAGATTGGATCTTGCTGGCAC |
| NCOA3 | GGGTTCCCTAAGGGTTGGACCATGTAAGATCAAAATACTTGCTGGATGGTGGACT | CAGAGACCAATAAAAATAAACTGCTTGAACATCCTTTGATTCATGAGTCTAGATTGGATCTTGCTGGCAC |

## Slide 11
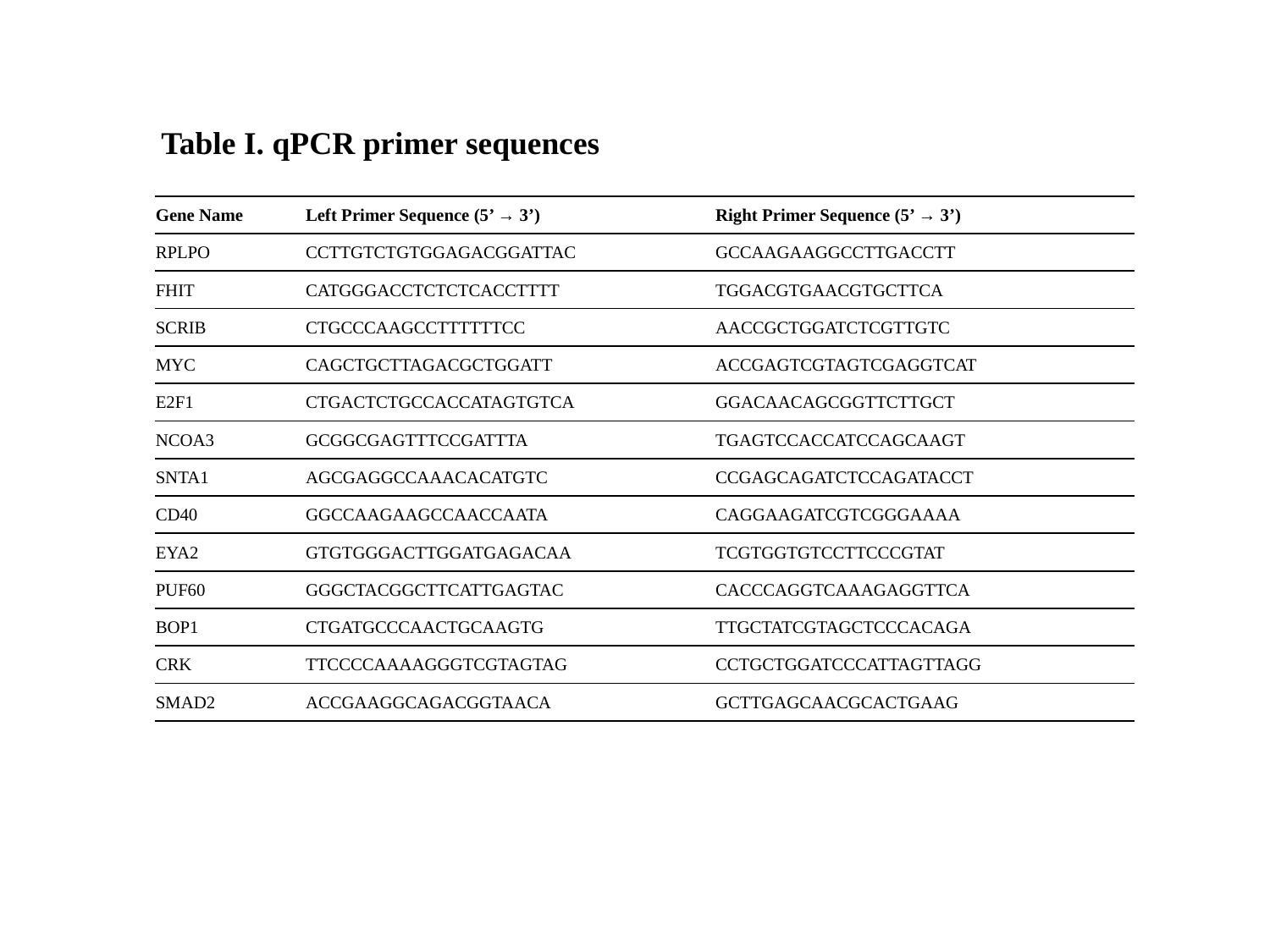

Table I. qPCR primer sequences
| Gene Name | Left Primer Sequence (5’ → 3’) | Right Primer Sequence (5’ → 3’) |
| --- | --- | --- |
| RPLPO | CCTTGTCTGTGGAGACGGATTAC | GCCAAGAAGGCCTTGACCTT |
| FHIT | CATGGGACCTCTCTCACCTTTT | TGGACGTGAACGTGCTTCA |
| SCRIB | CTGCCCAAGCCTTTTTTCC | AACCGCTGGATCTCGTTGTC |
| MYC | CAGCTGCTTAGACGCTGGATT | ACCGAGTCGTAGTCGAGGTCAT |
| E2F1 | CTGACTCTGCCACCATAGTGTCA | GGACAACAGCGGTTCTTGCT |
| NCOA3 | GCGGCGAGTTTCCGATTTA | TGAGTCCACCATCCAGCAAGT |
| SNTA1 | AGCGAGGCCAAACACATGTC | CCGAGCAGATCTCCAGATACCT |
| CD40 | GGCCAAGAAGCCAACCAATA | CAGGAAGATCGTCGGGAAAA |
| EYA2 | GTGTGGGACTTGGATGAGACAA | TCGTGGTGTCCTTCCCGTAT |
| PUF60 | GGGCTACGGCTTCATTGAGTAC | CACCCAGGTCAAAGAGGTTCA |
| BOP1 | CTGATGCCCAACTGCAAGTG | TTGCTATCGTAGCTCCCACAGA |
| CRK | TTCCCCAAAAGGGTCGTAGTAG | CCTGCTGGATCCCATTAGTTAGG |
| SMAD2 | ACCGAAGGCAGACGGTAACA | GCTTGAGCAACGCACTGAAG |
